# Scalable and compact photonic neural chip with low learning-capability-loss

**DOI:** 10.1515/nanoph-2021-0521

**Published:** 2021-12-22

**Authors:** Ye Tian, Yang Zhao, Shengping Liu, Qiang Li, Wei Wang, Junbo Feng, Jin Guo

**Affiliations:** Chongqing United Microelectronics Center (CUMEC), No. 20 Xiyuannan Road, Chongqing 100290, China; School of Information and Electronic Engineering, Hunan City University, Yiyang, 413000, China

**Keywords:** neural network, photonic computation, silicon photonics

## Abstract

Photonic computation has garnered huge attention due to its great potential to accelerate artificial neural network tasks at much higher clock rate to digital electronic alternatives. Especially, reconfigurable photonic processor consisting of Mach–Zehnder interferometer (MZI) mesh is promising for photonic matrix multiplier. It is desired to implement high-radix MZI mesh to boost the computation capability. Conventionally, three cascaded MZI meshes (two universal *N* × *N* unitary MZI mesh and one diagonal MZI mesh) are needed to express *N* × *N* weight matrix with *O*(*N* ^2^) MZIs requirements, which limits scalability seriously. Here, we propose a photonic matrix architecture using the real-part of one nonuniversal *N* × *N* unitary MZI mesh to represent the real-value matrix. In the applications like photonic neural network, it probable reduces the required MZIs to *O*(*N*log_2_ *N*) level while pay low cost on learning capability loss. Experimentally, we implement a 4 × 4 photonic neural chip and benchmark its performance in convolutional neural network for handwriting recognition task. Low learning-capability-loss is observed in our 4 × 4 chip compared to its counterpart based on conventional architecture using *O*(*N* ^2^) MZIs. While regarding the optical loss, chip size, power consumption, encoding error, our architecture exhibits all-round superiority.

## 1 Introduction

**Figure 1:**
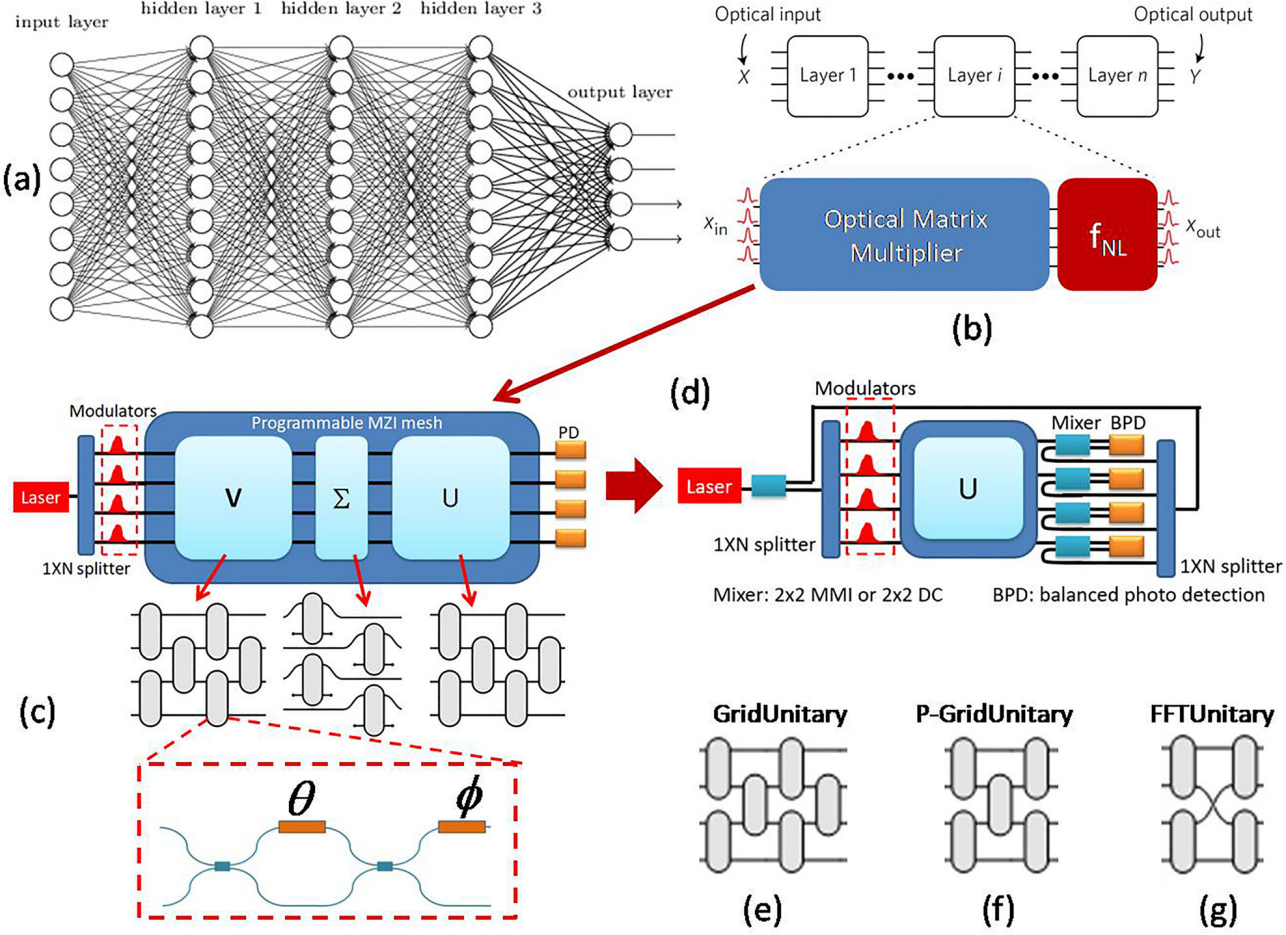
The schematic illustrition of the SVD mesh and pseudo-real-value mesh for matrix-multiplier in PNN. (a) General artificial neural network architecture composed of an input layer, a number of hidden layers and anoutput layer. (b) Decomposition of the general neural network into individual layers implemented by the optical matrix multiplier and nonlinearity units that compose each layer of the artificial neural network. Most operations are the performed on the optical matrix multiplier, which conventionally can be realized by (c) SVD-mesh containing *N* ^2^ 2 × 2 MZIs. (d) Schematic illustration of the pseudo-real-value matrix unitary MZI mesh for matrix expression; a schematic of (e) universal GridUnitary mesh, (f) nonuniversal P-GridUnitary mesh, and (g) nonuniversal FFTUnitary mesh. (Meaning of the abbreviation in figure, PD: photo diode; MMI: multimode interference; DC: directional coupler.)

Neural-like computation is desired because the neural system possesses much higher performance and lower energy consumption than current computers based on Von Neumann architecture for a wide range of tasks like perception communication, learning and decision making [1], [2], [3]. Motivated by these superiorities of the neural-like computation, artificial neural network (ANN, Figure 1a) is becoming increasingly attractive as a powerful tool to solve a large class of problems from face recognition to natural language processing [1], [2], [3], [4], [5]. However, the implementation of ANN is an ultra-computationally expensive task. Especially, it requires dense matrix computation [1, 6, 7]. Conventional digital electrical instantiation of matrix unit typically suffer from high communication overheads, expensive digital multiply-accumulate operations (MAC), high latency [6, 7]. Moreover, due to the slow-down of the size shrinking of the transistor driven by Moore’s Law, the performance of the digital matrix unit is approaching its physical-limitation, and become increasingly difficult to fulfill the needs of the swift-developing ANN technology on computation speed and power efficiency brought by larger model size and bigger data volume [1]. Alternatively, photonic processing is suggested as a candidate beyond Moore’s Law for dense matrix computation with stark advantages in bandwidth density, latency [6], [7], [8], [9]. As such, it is becoming increasingly attractive that photonic neural network (PNN, Figure 1b) can obtain lower energy cost but at much higher clock rate to the digital electronic counterpart, and several implementations, including coherent nanophotonic circuits, diffraction optics, photonic comb, etc., are developed [4, 5, 8, 10], [11], [12], [13], [14], [15].

Practically, among a plenty of photonic processing architectures [5, 8, 12, 13, 15], there has been much progress towards “universal linear optics” architecture: photonic circuits that can be defined to execute all possible linear optical matrix transformations on a given set of input modes [8, 16]. Such systems are typically built using planar meshes of beam splitters, which are easy to fabricate and to individually control [8, 17]. Basically, the optical matrix multiplier based on such mesh can be deemed as the cascaded interconnections of several 2 × 2 MZIs (Figure 1c) with some types of geometrical topology to realize unitary linear transformation [16], [17], [18]. Furthermore, utilizing the well-known singular value decomposition (SVD) algorithm (Figure 1c) [5]:

(1) $$E_{\text{out}}=ME_{\text{in}}\;where\;M=U\Sigma V$$

i.e., arbitrary real-value matrix multiplier can be factorized by two unitary matrixes *U*,*V* expressed by MZIs using Reck’s (or Clement’s) scheme, and a diagonal matrix Σ that can also be represented by MZIs. Such that the computationally expensive MAC can be operated on optical domain. However, at least *N* ^2^ 2 × 2 MZI cells are needed to realize arbitrary *N* × *N* matrix (Figure 1c). The square increase of the MZI number versus matrix dimension strongly limits the scalability of such architecture. It is possible to substitute the universal unitary mesh with nonuniversal one requiring less MZIs to improve the scalability [17, 19], but the cost is the impaired matrix expressivity, and accordingly degraded learning-capability in implemented PNN [17].

Moreover, due to the diffraction-limitation, the photonics device is almost impossible to shrink its size like the electrical device (e.g., the transistor) beneficial from upgrading the process node. The plasmonic devices might break the diffraction limitation and achieves subwavelength dimension [20], [21], [22], [23], but so far, they are too lossy to integrate on large scale. Hence, large scale photonic circuit integration would be restricted by the fast expansion of the chip size, e.g., Lightmatter’s Mars device integrated 64 × 64 MZI with nano-optical-electro-mechanical-system as the phase-shifting elements on a 150 mm^2^ chip, and accordingly the predicted chip size for 1024 × 1024 would be prohibitively high as 384 cm^2^ and difficult to realize [19]. Besides, the energy efficiency of current photonic matrix unit utilizing thermal or electrical phase shifter seems not as low as expected, e.g., the power required to maintain the state of the MZI by thermal-optical phase shifter is typically from few to tens mW per shifter [6, 7, 24], which, in some cases, may account for ∼87% of total energy consumption in an intact photonic computing system [24]. Hence it is rather challenging for presented photonic processing architecture to copy the success of Moore’s law on electronic computing to upgrade the performances by reducing the device size as well as consumed power. Whereas, it is more significant to exploring superior architecture to overcome the scale-limitation of current experimental demonstrated PNN as well as by-produced bottleneck on chip size and energy consumption.

In this article, we propose a scalable architecture with reduced MZIs requirement least at *O*(*N*log_2_ *N*) level. Other than regular mesh design containing *N* ^2^ MZIs to construct two universal unitary matrixes *U*, *V* and one diagonal matrix Σ given by SVD algorithm to express arbitrary N*N real-value matrix, our scalable design, namely, pseudo-real MZIs mesh employs the real-part of an unitary mesh to learn the real-value matrix, (i.e., the mesh itself is truly programmed as a complex-value matrix, but works as a real-value matrix multiplier whose operation only depends on its real part). Theoretically, our method could construct 2^*N*^ available unitary matrixes whose real part is exactly the desired real-value matrix to learn. Hence, a nonuniversal mesh with few MZI cells might be good enough only if one of these 2^*N*^ available unitary matrixes can be expressed by this nonuniversal mesh. Such that the pseudo-real-value mesh could reduce required MZI cells (thus lower power and smaller chip size) to provide better scalability to high radix. In the applications like PNN, we use the most compact design consuming only 0.5*N*log_2_ *N* MZIs, pseudo-real-value FFTUnitary MZI mesh, to show the feasibility to pay low cost on accuracy loss while apparently reduce the required photonic devices. Experimentally, we implement an 4 × 4 photonic neural chip and benchmark its performance in convolutional neural network for handwriting recognition task, which achieve the accuracy fully comparable to regular SVD-mesh as well as current digital computer.

## 2 Principles of pseudo-real-value PNN

The key thought of our design is that we do not try to find the expressions to two universal unitary matrix *U*,*V* and a diagonal matrix Σ separately as done in conventional SVD-based method to represent given real-value matrix *M*, but construct a matrix *U* whose real part fulfill that

(2) Re(U)=αM

and then the real part of the output *E*_out_, which is produced from the amplitude-modulated input modes *E*_in_ multiply with *U*, would be equivalent to the target multiply-operation *ME*_in_ with only a difference of coefficient *α*, i.e.,

(3) Re(Eout)=Re(UEin)=Re(U)Ein=αMEin

where the extraction of the real part of the output can be simply realized by interfering the output with a fixed reference source light. As such, infinitely many complex matrixes *U* satisfying Eq. (3) could be used for the target multiply-operation since the image part of *U* could be arbitrary. This is why we call our design as pseudo-real MZIs mesh (i.e., the designed *U* itself is a complex-value matrix unit, but truly enables a real-value matrix multiplier operation depending on its real part). Consequently, even a less-expressive mesh might be good enough only if it could effectively approach one of the (infinitely many) available matrixes.

Specified as shown Figure 1d, let us set the light source as two parts, one part with a ratio *µ* of the total intensity of the input light source *I*_in_ is used for excite the intensity modulated input modes as done in conventional SVD-mesh-based analog-matrix multiplication. The other part, i.e., (1 − *μ*)*I*_in_ is used as the reference sources to interfere with the output modes. As such the interference between the reference sources *E*_ref_ and output modes *E*_out_ produce homodyne signals as

(4) $$I_{\pm }={\left|E_{\text{ref}}\right|}^{2}+{\left|E_{\text{out}}\right|}^{2}\pm \left|E_{\text{ref}}\|E_{\text{out}}\right|\text{cos}\left(\phi_{\text{out}}-\phi_{\text{ref}}\right)$$

where *ϕ*_ref_ and *ϕ*_out_ are the phase of reference sources and output modes. So the balanced detection to the homodyne signals allows us to extract the real part of the output if setting the initial phase of the reference sources as 0 (see SI-1 for further discussions):

(5) $$\begin{array}{l} I=I_{+}-I_{-}=2\left|E_{\text{ref}}\|E_{\text{out}}\right|\text{cos}\left(\phi_{\text{out}}\right)\\ =2\left|E_{\text{ref}}\right|\text{Re}\left(E_{\text{out}}\right)\\ \propto 2\sqrt{\mu \left(1-\mu \right)I_{\text{in}}}ME_{\text{in}} \end{array}$$

i.e., when the input modes are purely amplitude-modulated, the real part of the output modes can be seen as the multiplication of the input with the real part of the matrix represented by the MZIs mesh other than the matrix itself as done in conventional methods [5, 8, 16, 17]. Hence no matter what the image part of the matrix expressed by the MZIs mesh is, if Re(*U*) is exactly the target real-value matrix *M* we would like to define, actually we have executed the desired matrix multiplication according to Eq. (3). Besides, unlike the regular intensity-detection scheme whose photonic matrix elements is always positive, here the pseudo-real MZI mesh architecture intrinsically support both positive- and negative-value calculation. Furthermore, since Im(*U*) is arbitrary, we can set the Im(*U*) as that fulfill the property of *U* = Re(*U*) + *j*Im(*U*) and at the same time *U* is a unitary matrix. Therefore, other than using two unitary matrixes and one diagonal matrix need in conventional SVD-based mesh design (Figure 1c), one unitary matrix is enough to implement any given matrix multiplication only if we have a general algorithm to construct the unitary matrixes whose real part are just the target real-value matrix. The desired algorithm is as following:

1) Decompose the target real-value matrix by SVD as *M* = *βUΣV* [17], here *β* could be a parameter depending on light power, mesh loss and detection gain, which is rational configured to ensure every singular value *σ*_*ii*_ (*i* = 1, 2, … *n*) in diagonal Σ is positive but less than 1, while *U* and *V* could be two real-value unitary matrix. Without the loss of the generality, we choose *β *=* *1 in the following to simplify the discussion. Take the case of matrix radix *N* = 4 as an instance, we have

(6) $$\begin{array}{l} M=U\Sigma V\\ \text{where }\Sigma =\left[\begin{array}{llll} \sigma_{11} & 0 & 0 & 0\\ 0 & \sigma_{22} & 0 & 0\\ 0 & 0 & \sigma_{33} & 0\\ 0 & 0 & 0 & \sigma_{44} \end{array}\right] \end{array}$$

2) We mathematically construct a new matrix *M*′ = *UΣ*′*V* whose singular value *σ*_*ii*_′ (*i* = 1, 2, … 4) is correlated to the count part in *M* with ${\left(\sigma_{ii}\right)}^{2}+{\left(\sigma_{ii}^{\prime }\right)}^{2}=1$ .
3) The matrix *M* + *iM′* would be a designed unitary matrix *U*_D_ with *M* = Re(*U*_D_), if noting (*M* + *iM′*) (*M* + *iM′*)^*H*^ = *U*(Σ + *iΣ*′)*VV* ^*H*^(Σ + *iΣ*′)^*H*^ *U* ^*H*^ = *I* (here, *I* is the identity matrix). Hence the pseudo-real mesh design could achieve the minimal optical depth, requiring less than half the depth of the SVD-based design – other than three MZI mesh for *U*, Σ and *V*, only one unitary mesh *U* used to express *M* + *iM′* is enough. This seems valuable for minimizing optical losses and reducing fabrication resources [16]. For example, based on the MZI unit cell shown in Figure 1c, the GridUnitary mesh (Figure 1e) has universal expressivity to any unitary matrix *via* Clement’s algorithm [5, 25]. Accordingly, when using pseudo real-value design with GridUnitary mesh to implement arbitrary real value matrix [17], the needed MZI cells could decrease from *N* ^2^ in conventional SVD mesh design to *N*(*N* − 1)/2.

Furthermore, it is noteworthy that for each *σ*_*ii*_ and *σ*_*ii*_′, their sign could be either same or opposite. Therefore, for a given real value N*N matrix *M*, we can construct at least 2^*N*^ unitary matrixes whose real part equal *M*. That is to say, in principle, a nonuniversal mesh with few MZI cells, might actually have sufficient capability to represent any real value N*N matrix *M* when using pseudo-real mesh design only if one of these 2^*N*^ available unitary matrixes could be expressed by this nonuniversal mesh, e.g., a partial-GridUnitary mesh (P-GridUnitary, Figure 1f) built by removing the last stage of a standard (4 × 4) GridUnitary mesh or the FFTUnitary mesh (Figure 1g) proposed in [17, 26], while the performance degradation due to the usage of less-expressive unitary mesh in conventional SVD-based methods might be highly relieved [17, 25].

## 3 Learning-capability of the pseudo-real-value architecture

Matrix-vector-product (MVP) is the most frequently used and computationally expensive operations in the neural network algorithms [13, 15, 27]. Hence in this section, we study the real-value matrix expressivity of aforementioned pseudo-real-value design with universal and nonuniversal MZI mesh at first. Afterward, considering for the specific tasks, the primary figure of merit is the classification or detection accuracy but not the matrix-expressivity accuracy, we study their performances in the specific neural network models for different dataset to evaluate the learning capability loss to using pseudo-real-value nonuniversal unitary mesh for PNN tasks.

### 3.1 Real-value matrix expressivity

#### 3.1.1 Numerical evaluations for small radix matrix

**Figure 2:**
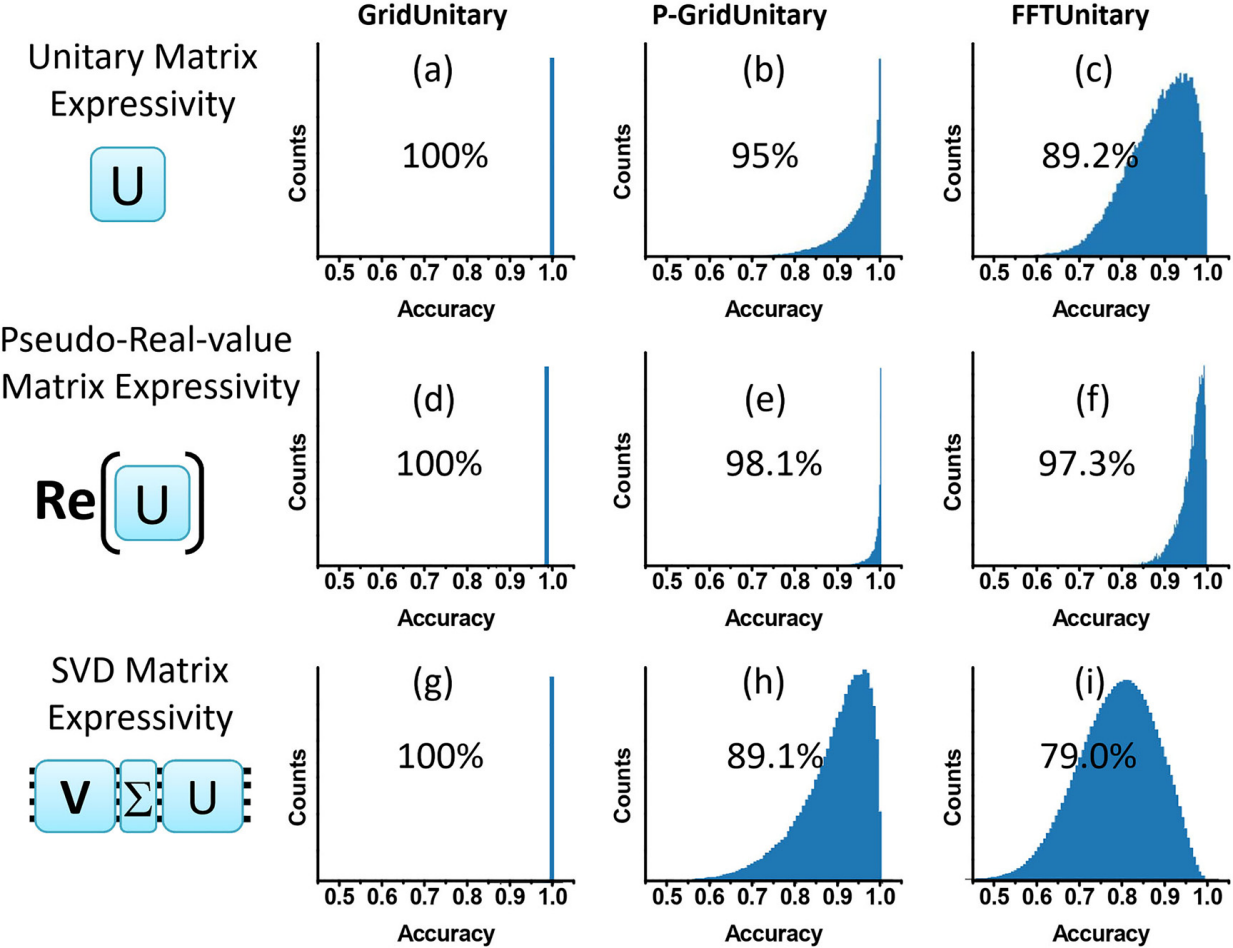
The statistical accuracy distribution of expressing 4*4 unitary matrix by (a) GridUnitary mesh, (b) P-GridUnitary mesh, and (c) FFTUnitary mesh; the statistical accuracy distribution of expressing real-value matrix by real-part of (d) GridUnitary mesh, (e) P-GridUnitary mesh, and (f) FFTUnitary mesh. The statistical accuracy distribution of expressing real-value matrix by conventional SVD matrix with (g) GridUnitary mesh, (h) P-GridUnitary mesh, and (i) FFTUnitary mesh as the unitary matrix operator.

We numerically study the matrix expressivity of aforementioned pseudo-real-value design with universal and nonuniversal MZI mesh. Exactly, due to the lack of effective analytical or numerical decomposition algorithms for nonuniversal mesh like P-GridUnitary and/or FFTUnitary mesh to approach given unitary matrix, it is difficult to analytically obtain the global optimal for the pseudo-real nonuniversal mesh to express arbitrary random sampled real-value matrix [17]. Even though, for small matrix dimension *N* (*N* = 4), the mature methods in machine learning like stochastic gradient descent (SGD) still achieve satisfying local optimal. Specified, we generate 10,000 random arbitrary target 4 × 4 real matrices, and each real matrix could be the real part of at least 2^4^ = 16 kinds of unitary matrices, accordingly it would produce 160,000 available unitary matrices. Afterward, we use the GridUnitary, P-GridUnitary and FFTUnitary mesh to approach these 160,000 unitary matrices *via* SGD method (SI-2), and, respectively, founds 100, 95.2 and 89.1% average accuracy (defined as Tr(Re(*U*_tar_*U_app_^*H*^)), where *U*_tar_ is the available unitary matrix as the target, *U*_app_ is the obtained unitary matrix approached by SGD) as shown in Figure 2a–c. But, considering only one unitary-matrix whose real part is equal to the target real matrices is enough, we practically choose the best one from 16 corresponding approached unitary-matrices as the final solution. Such that, the statistical accuracy to approach the random arbitrary target real matrices by pseudo-real-value matrix with nonuniversal P-GridUnitary and FFTUnitary mesh design would increase to 98.1 and 97.3%, much better than the performance of P-GridUnitary and FFTUnitary mesh themselves on approaching unitary matrices (Figure 2d–f). In addition, as a baseline, these 10,000 random 4 × 4 real matrices can be also learned by conventional SVD architecture. Considering for regular SVD mesh, the diagonal matrix Σ can be always learned correctly, the learning-performances is fully dependent on the expressivity of the universal or nonuniversal unitary mesh on unitary matrix *U* and *V*. Accordingly, for an given matrix, we use Tr(Re(*U*_tar_*U_app_^*H*^)*Tr(Re(*V*_tar_*V_app_^*H*^) to evaluate the performances of SVD architecture with universal GridUnitary, or nonuniversal P-GridUnitary and FFTUnitary mesh as shown in Figure 2g–i, which, respectively, produces 100, 89.1 and 79.0% average accuracy. That is to say, in the conventional SVD-architecture (Figure 1c), which contains two unitary mesh, substituting the universal unitary meshes to nonuniversal ones would highly impair the expressivity to the real-value matrix due to their remarkable lower fidelity. Whereas, for the pseudo-real-value matrix design, the accuracy-loss brought by using the nonuniversal unitary mesh to represent the real-value matrix is apparent less because of the much amplified feasible solution space. Moreover, considering the high error tolerance of ANN allowing inference at low precision of 4–5 bit [28], [29], [30], even for the 4 × 4 FFTunitary mesh with least MZIs (only 25% MZIs consumption comparing to universal 4 × 4 SVD mesh with 16 MZIs), the expressivity error of ∼2.7% is still lower than the quantization error. Besides, the nonuniversal unitary mesh has shallower optical depth, which is important to minimize optical losses and enhance the robustness to the fabrication imperfection [25], as well as reduce calibration complexity.

**Figure 3:**
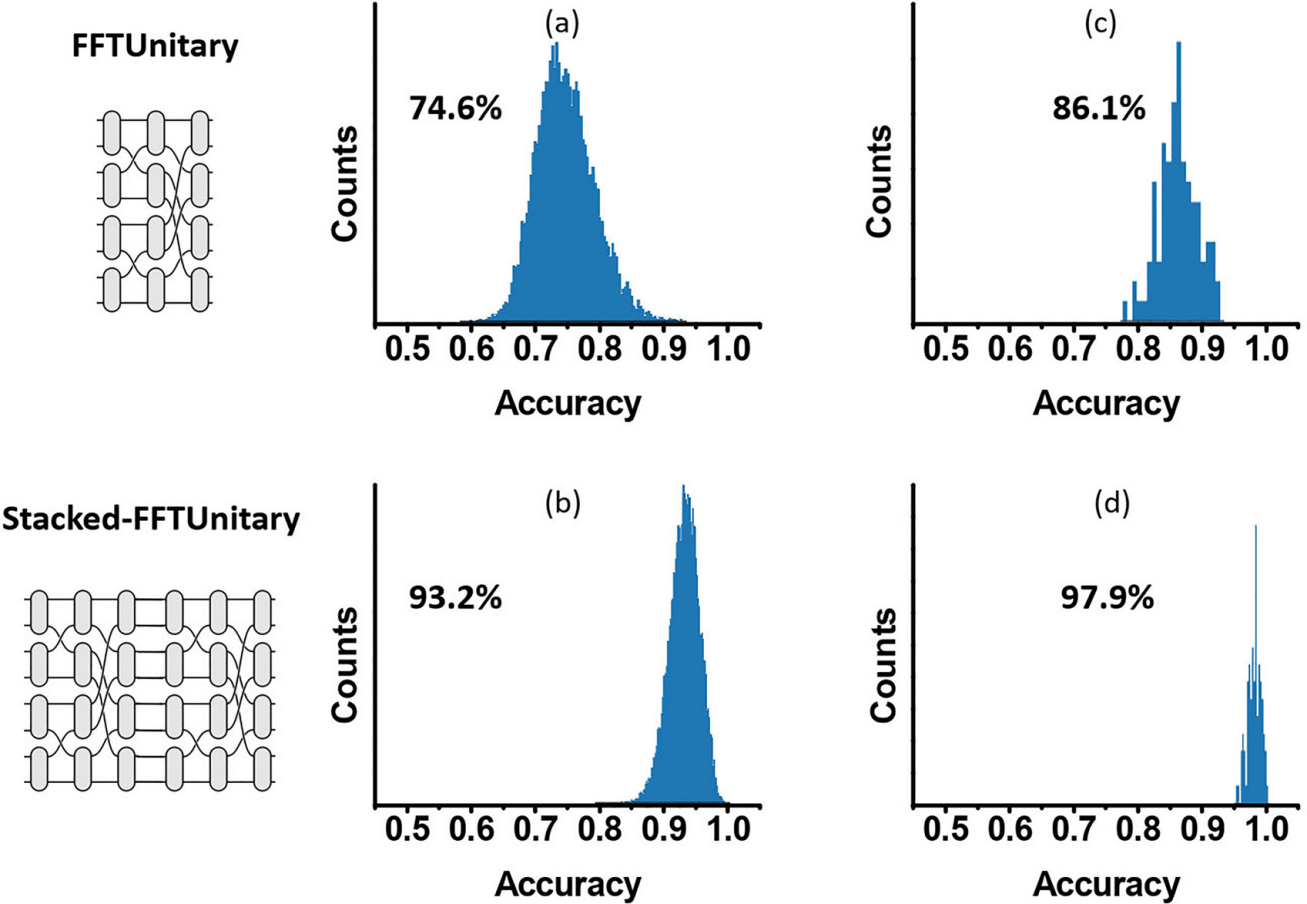
The statistical accuracy distribution of expressing 8*8 unitary matrix by (a) FFTUnitary mesh and (b) stacked-FFTUnitary mesh; the statistical accuracy distribution of expressing minimal-error one picked for real-value matrix based on pseudo-real-value (c) FFTUnitary mesh and (d) stacked-FFTUnitary.

It seems promising of the pseudo-real-value architecture to reduce the MZIs requirement and accordingly improve the scalability while maintain satisfying learning capability for radix *N* = 4. To further check the convergence property of the proposed pseudo-real-value optimization approach, we explore the availability on larger radix. To simplify the discussion, we focus on the pseudo-real-value architecture with FFTUnitary mesh or its stacking (Stacked-FFTUnitary mesh), because FFTUnitary mesh consumes the minimal number of MZIs at *O*(N*log_2_ *N*) level to make all input modes interact with each other and consequently might approach arbitrary unitary matrices [31]. Hence, the pseudo-real FFTUnitary mesh might be a promising candidate with most effective-use of the MZIs, while the intrinsic advantage of FFTUnitary mesh on fabrication imprecision tolerance maintains [17]. To ensure log_2_ *N* is an integer, *N* should be 4, 8, 16, etc. Hence, as the extension to aforementioned study to *N* = 4 case, the expressivity of 8*8 FFTUnitary and Stacked-FFTUnitary consists of FFTUnitary multipliers stacked end-to-end 2 times are investigated as shown in Figure 3. These two meshes are both nonuniversal and consume MZIs less than the universal one. It found that 8*8 FFTUnitary and Stacked-FFTUnitary mesh could, respectively, achieve 74.6 and 93.1% accuracy on learning 25,600 unitary matrix constructed from 100 8*8 random real-value matrixes (Figure 3a and b), and the minima-error one picked from 256 candidates could optimize the statistical accuracy to 86.1 and 97.9% (Figure 3c and d, although the distribution profile is not very smooth due to limited 100 sample times). Therefore, the results of both 4*4 and 8*8 matrixes support the feasibility of using pseudo real-value nonuniversal mesh with *O*(N*log**** *N*) MZIs to learn real-value matrix accurately. For both *N* = 4 and *N* = 8 cases, it is feasible to use reduced MZIs (4 cells and 24 cells, respectively) to achieve good-learning (e.g., accuracy > 97%) comparable to conventional SVD design (16 cells and 64 cells, respectively), indicating the improved scalability of the pseudo-real-value nonuniversal unitary mesh architecture with low matrix expressivity loss. However, the learning for cases of *N* = 16 or more becomes quite time-consuming on our computer (SI-2): as *N* increases, the learning time would grow exponentially since we need to train 2^*N*^ times to traverse the approaches of the given mesh to all available unitary matrices and range their representation error to pick out the minimal-error one. In future, it is quite desired to develop more efficient approaching methods.

Although the picking process consumes exponentially-increased training time and becomes not so practical for large *N*, the fidelity distribution for the mesh to learn arbitrary unitary matrix allow us to track the mathematic expectation of the minimum error under some rational assumptions. Here, we introduce a fidelity-dependent error *E*_F_: *E*_F_ = 1 − fidelity. Then the picking process proposed above would be something like estimating the mathematic expectation of the minimum of the 2^*N*^ samples for the random variable following *E*_F_ distribution, whose profile can be tracked from numerically simulated fidelity distribution. When the mesh is with few layers (e.g., 8*8 FFTUnitary case shown in Figure 3a), the approaching is equivalent to embedding the *N* × *N* matrix into a (small) N*log_2_**** *N* subspace, hence the (large) ∼2^*N*^ sampling times imply that the distribution should be approximately Gaussian according to central-limit-theorem. But for the mesh with more layers (e.g., 8*8 Stacked-FFTUnitary case shown in Figure 3b), *E*_F_ distribution is asymmetric with a most probable value relative close to low-error (high fidelity) side. Therefore, the mathematic expectation of the minimum sampled value for such distribution should be lower than the counterpart calculated for Gaussian distribution. Even though, the error-expectation calculated for Gaussian distribution is still a good estimation to its upper bound. Likewise, the calculation for uniform distribution could restrict the lower-bound of the error-expectation (SI-3). This might be valuable for us to understand the expressivity of the mesh with very large *N* considering the difficult on numerical simulating the matrix-learning at high radix through existing minima-error picking route.

#### 3.1.2 Discussions to the matrix expressivity at large-*N* limitation

Although it is challenge to numerical-study the matrix expressivity of pseudo-real value nonuniversal unitary mesh by aforementioned minima-error picking method at large radix *N*, the qualitative discussions to this issue are still of significance. More specified, how about the least MZIs needed for a nonuniversal unitary mesh to produce good enough expressivity to arbitrary dense (real-value) matrixes? And furthermore, since the weight kernels (matrixes) used in neural network applications concerned in this work usually tend to be sparse compare to random data and probable embedded to low-dimensional spaces [32], it is also of interest to see how about the least MZIs needed for a nonuniversal unitary mesh to express sparse matrix?

For the N*N matrix multiplication based on MZI mesh, it is physically implemented by feeding in *N* sequential 1 × *N* column vector into the mesh and detect the outputs. Accordingly, if the MZI used by the mesh is *n*_MZI_, then the operation number to complete the matrix multiplication on optical domain should be on *O*(*n*_MZI_*N) level. Obviously, on the viewpoint of the computation complexity, we have:

(7) $$O\left(N^{2}\right)<O\left(n_{\text{MZI}}*N\right)<O\left(N^{3}\right)$$

where *O*(*N* ^3^) is given by the naive (‘schoolbook’) algorithm, and *O*(*N* ^2^) is based on the fact that any algorithm must output *N* ^2^ entries. Consequently,

(8) $$O\left(N\right)<O\left(n_{\text{MZI}}\right)<O\left(N^{2}\right)$$

It is straightforward that based on Reck and Clemamnts’s method, the pseudo-real-value architecture proposed here can use *N*(*N* − 1) MZIs to represent arbitrary dense (real-value) matrixes [15, 18, 33]. However, the advances on optimizing the computation complexity of the matrix multiplication may allow us see more. Very recently, the computational complexity of the multiplication between two N*N matrices was optimized to from *O*(*N* ^3^) to *O*(*N* ^2.3728595^) by Alman and Williams [34], indicating the possibility to accordingly approach arbitrary dense matrix with sub-*O*(*N* ^2^) MZIs (e.g., we may achieve *O*(*N* ^1.3728595^) circuit complexity *via* photonic mapping to such optimized algorithm of the matrix multiplication). Moreover, Ran Raz proved a lower-bound to the computational complexity of the matrix multiplication as *O*(*N* ^2^log**** *N*) [35], but no one has developed the algorithm to reach this bound so far. However, if relax the requirement from dense matrix to sparse matrix, Likhosherstov et al., from Google demonstrate the feasible approaching [32]: They theoretically proved based on the Johnson-Lindenstrauss lemma [36], and experimental verified the log-dimension embedding for the expressive power of (sparse) self-attention matrices. As such, the sparse matrix might be approximated by mesh with the least MZIs at *O*(N*log**** *N*) level. Based on these analysis, we further suggest the relationship between the matrix expressivity and the required MZIs of the pseudo-real-value mesh (SI-4), which may explain why the mesh with *O*(N*log**** *N*) MZIs could approach arbitrary real-value matrix with small radix (e.g., *N* = 4 and 8) accurately as shown above (Figures 2f and 3d), but would degrade to just feasible for sparse matrix in the large-*N* limit as found in [32]. Anyway, further investigations to the matrix expressivity of pseudo-real-value nonuniversal unitary mesh are still highly desired.

### 3.2 PNN training on pseudo-real-value nonuniversal mesh

It has been well known that an *N* × *N* MZI mesh representing real-value matrix requires *O*(*N* ^2^) MZI cells and *O*(*N*) cascaded stage based on conventional SVD scheme, highly limiting the scalability of this architecture to high radix. However, considering in the applications like the classifications based on ANN, rather than the optimization toward the simulation of a specific matrix, the linear operation learned from the classification task is not, *a priori*, known. Therefore, the more primary figure of merit is the classification accuracy instead of the fidelity between the target unitary matrix and the one learned [17], and the nonuniversal mesh may also perform well. This is because machine-learning features tend to be low-dimensional compared to random data [32, 36]. In several practices, the methods, including dropout [37], pruning [38], etc., had been employed to reduce the operations of the neural networks without significant degradation in accuracy. Actually, the aforementioned results imply that it is feasible to the reduce the needed MZI cell by using pseudo-real-value matrix with nonuniversal mesh design without remarkable learning-capability-loss at small radix *N* (e.g., 4*4 FFTUnitary and 8*8 Stacked-FFTUnitary), and even in large-*N* limit, the mesh with MZIs reduced to *O*(N*log**** *N*) level might be still good enough to express the weight kernels (or self-attention matrix) in ANN which can often be low-demission-embedded [32]. That is to say, the MZIs in reduced-depth mesh are probable effective-used for PNN, even if the expressivity of meshes is not able to cover arbitrary matrices in larger radix limit. Hence, in this section, we design scalable and compact PNN based pseudo-real-value nonuniversal unitary mesh and numerically evaluate their performances.

Here, two types of neural networks are considered, LeNet-5 and MobileNet. The former is used to MNIST dataset, while the latter is trained for relative complex Fashion-MNIST dataset. The LeNet-5 networks is with two hidden layers, respectively, containing six 3*3 convolution kernels and ninety-six 4*4 convolution kernel, and three full-connection layers (Figure 3a inset, see further details in SI-4). Since all the convolutional kernels are 3*3 or 4*4, we use the 4*4 pseudo-real-value universal (4*4 GridUnitary) or nonuniversal mesh (4*4 P-GridUnitary or 4*4 FFTUnitary) as the matrix multiplier within, while the network using conventional universal SVD-GridNet mesh is used as the baseline. While in MobileNet, there are several kernels suitable to executed by matrix multiplier with large radix, hence we use several different large N*N pseudo-real-value matrix with FFTUnitary meshes (SI-5), which is the most compact nonuniversal unitary mesh design having all input modes interact with each other and consequently might approach arbitrary unitary matrices [39, 40]. As such, accounting different network models, different dataset, and different radix of the matrix multiplier, the evolution to the potential of pseudo-real-value nonuniversal unitary mesh could be more robust.

The LeNet-5 is trained for 40 training epochs. Afterward, the pseudo-real P-GridUnitary mesh and FFTUnitary mesh achieves the classification accuracy of 99.33 and 99.35%, respectively, almost the same to the case of universal GridUnitary mesh (99.36%) as shown in Figure 3a–d, which is used as the baseline for comparison, further verifying their closeness on real-value matrix expressivity as shown in Figure 2. Moreover, for the conventional SVD-based universal mesh, considering the matrixes (or convolution kernel) in trained network model can be decomposed to *U*,*V* and a diagonal matrix Σ (Eq.(1)), and then the phase-shifters setting to produce these three multiplier can be obtained by the scheme from Reck or Clements et al. [17, 33], we may use the network directly trained in real-value domain to benchmark the performance of the conventional SVD-based universal mesh. Interestingly, the pseudo-real mesh perform better than conventional SVD-based mesh which achieves relative lower network accuracy of (98.77%, Figure 3a and e), in line with the observation in previous reports [17, 27]. We suggest that when the conventional SVD-based mesh and pseudo-real MZI mesh have similar real-value matrix expressivity, the pseudo-real architecture might be intrinsically easier to train. Perhaps this is because its much less phase parameters lead to smaller parameter space needed to search and accordingly higher possibility to reach the optimum after the same training epoch.

For the case of MobileNet using high-radix pseudo-real-value FFTUnitary mesh (SI-4), it also achieves acceptable ∼89.4% accuracy for 10,000 test instances of the relative complex Fashion-MNIST dataset (Figure 5a), which is slightly lower than the baseline of ∼92.3% with universal matrix multiplier (Figure 5a–c), indicating the feasibility to pay acceptable cost on learning capability loss to apparently reduce the photonic devices consumption. Such superiority of the FFTUnitary mesh was also observed in the recurrent neural network applications of efficient unitary neural networks (EUNN) [31], where the EUNN using FFTUnitary mesh (radix = 128) shows slightly lower but quite close performances in compare with the EUNN using universal unitary mesh on TIMIT dataset for real-world speech prediction. As such, for either small or large radix *N*, we suggest the pseudo-real-value FFTUnitary mesh could be always a reasonable candidate for photonic matrix multiplier with low accuracy loss on PNN tasks. Some better training methods, especially the ones suitable for FFT-like architectures may further narrow the performances gap between the nonuniversal and universal mesh designs [41], [42], [43].

## 4 Experimental implementation PNN on pseudo-real-value nonuniversal mesh

### 4.1 Setup and methods

Aforementioned pseudo-real-value MZI mesh can be implemented on silicon photonics chip, and execute the matrix multiplier operation at very high time clock, as well as realize ANN inference. Specified, as a beginning, here we construct an inference system based on a 4*4 pseudo-real-value MZI mesh, and implement a LeNet-5 network on this system for MNIST dataset classification. Before the detail discussions to the results, we briefly introduce the experimental setup and methods about the implementation.

#### 4.1.1 Chip fabrications

The chips are prepared by the Chongqing United Microelectronics Center (CUMEC) with its CSiP-180AL platform. The further details of the platform can be seen in https://service.cumec.cn/.

#### 4.1.2 Phase shifter characterization

The *I*–*V* characteristics of each heater on the phase shifter (PS) were tested by Keithley 2636A sourcemeter to get the resistance value. Afterward, the characterization of each PS was done by varying the applied voltage *V* while measuring the optical power at the output port. The collected measurement data were fitted with *y* = a*cos(b*P +* c*) + *d* to extract the power efficiency of the PS, where *y* was proportional to the photocurrent, *d* was a constant background, *a* was the maximum magnitude of the signal, *b* and *c* were coefficients depicting the relationship between the phase and the electrical power *P* computed by *P* = *V* ^2^ *R*. The typical *P_π_* of the shifters is ∼21.05 mW (SI-6).

#### 4.1.3 Implementation of the system based on pseudo-real-valued photonic neural chip

The light source was a 1550 nm laser with 9 dBm power (Optilab DFB-1550-PM-M-30). A polarization controller was applied to maximize the coupling of the light source to the chip. A home-built 96 channels voltage source consist of 6 pieces of 16-channels digital-to-analogue convertor (DAC, AD5767) and controlled by FPGA(XC7Z100FFG900-2) is employed to drive the thermal-optical modulator and configure the MZI mesh. The input data is encoding on the 4-channels modulator array by a home-built 4-channels DAC modules (AlINX, AN9767*2) configured by the FPGA. The output data is received by the on-chip balanced photo detector (BPD) with TIA (HMC799) and corresponding analogue-to-digital convertors (AlINX, AN9238*2). The input vector is purely amplitude-encoded by four modulators under differential-drive mode, and multiplied with the matrix expressed by the MZI mesh. After that, the output vector is obtained by four sets of BPD as the results of the MVP operations. The convolutions can be implemented though repeated MVPs. All the convolutions in our experiments are executed on the optical domain. A Thermo Electric Cooler (TEC) is employed to stabilize the chip-temperature at 25 ± 0.002 °C level to minimize the influence of the environmental temperature fluctuation. The system-level experimental precision of the pseudo-real-valued photonic neural chip on expressing these convolution kernels is ∼95% (SI-7). While the ReLU nonlinearity or activation as well as the rest full connection (FC) layers are carried out on electrical domain by FPGA.

#### 4.1.4 Photonic matrix multiplier based on the active chip at high time clock

To demonstrate the availability of the pseudo-real-value MZI mesh work on high speed, a silicon photonic chip integrating the PN-depletion-type MZM modulators working on differential drive mode, programmable 4 × 4 MZI mesh with pseudo-real-value design as well as the balanced photon detectors is employed. The commercial four-channel driver (Macom, MASC-37053A) is used feed in 25GBaud NRZ signals to the modulator, while the four-channel receiver chip (Macom, MATA-37244E) detect the output corresponding to the product of the input and the weight matrix expressed by the MZI mesh. As an example, here our chip executes the matrix multiplication of an identity matrix and the photonic matrix expressed by the MZI mesh, which (quantified to 1 bit) is

$$\left(\begin{array}{llll} 0 & 1 & 0 & 0\\ 0 & 1 & 0 & 0\\ 0 & 0 & 1 & 1\\ 1 & 0 & 0 & 0 \end{array}\right)$$

We input the 25GBaud NRZ signal (generated from arbitrary waveform generator Keysight-M8194A) sequentially from 1st, 2nd 3rd and 4th modulator. These four cases, respectively, represent the four column vectors of the identity matrix as (1,0,0,0)^T^, (0,1,0,0)^T^, (0,0,1,0)^T^ and (0,0,0,1)^T^, and can be verified by the eye diagrams of the four channels extracted from their corresponding monitor ports (tested by oscilloscope Keysight-N1092C). While the eye diagrams of the four output ports shows the results of the matrix-vector multiplication as four vectors, and they together form the results of aforementioned matrix multiplication (tested by oscilloscope Keysight-N1092C).

### 4.2 Photonic neural chip: experimental implementation and performance evaluation

**Figure 4:**
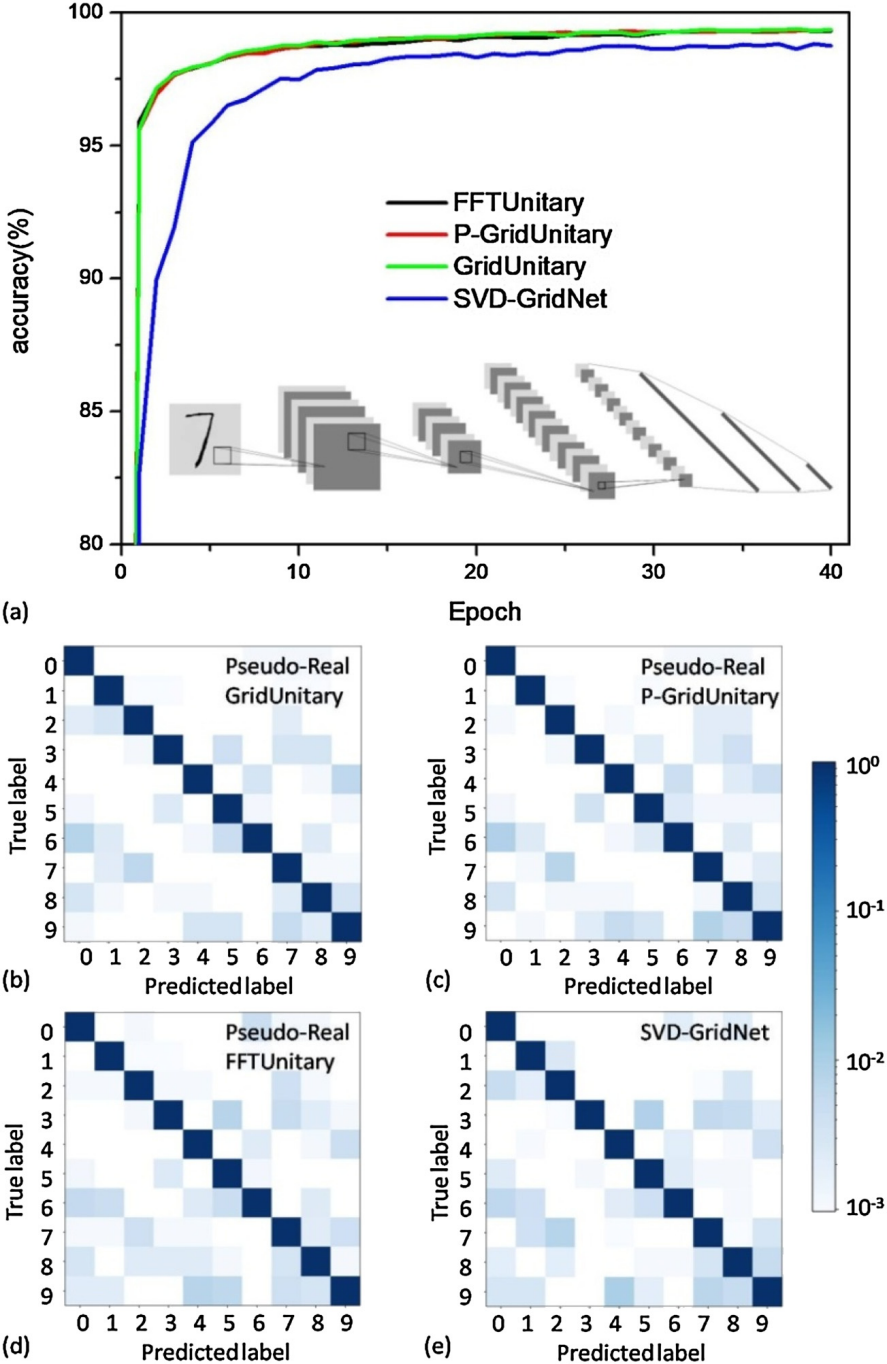
The performances of PNN on LeNet-5 model for MNIST task using pseudo-real-value GridUnitary, P-GridUnitary and FFTUnitary mesh, as well as their comparision to SVD GridNet. (a) The evolution of the MNIST accuracy of LeNet-5 model based on 4*4 pseudo-real GridUnitary (green), P-GridUnitary (red) and FFTUnitary mesh (black), as well as conventional SVD-GridNet mesh with 40 epoch training; confusion matrix of trained model on test set with 10,000 instances based on pseudo-real (b) GridUnitary, (c) P-GridUnitary and FFTUnitary mesh, as well as conventional SVD-GridNet mesh. Each column of the matrix represents the instances in predicted label, while each row represents the instances in true label.

The trained LeNet-5 network is implemented on an interference system based on silicon photonics chip including four-modulator array for (pure amplitude) data-input, pseudo-real-value matrix with P-GridUnitary mesh design and four balanced detector array to receive output-data. Such that all 3 × 3 and 4 × 4 kernels in convolution layers are performed on optical domain, while the nonlinear activations and full connection layers are executed in FPGA (Figure 6a–c). Experimentally, the interference system achieves 99.40% accuracy on MNIST test set including 10,000 instances (Figure 6d), while the MZI black dash marked in Figure 4c is configured to cross state, the P-GridUnitary mesh become equivalent to a FFTUnitary mesh, and then the test accuracy is 98.87% (Figure 6e). They are both very close to the training performance, indicating the low encoding error level of pseudo-real-value nonuniversal mesh for matrix expression. During the whole interference process, the classification accuracy is quite stable at high level without apparent deviation (see Video-1), whereas in conventional SVD-mesh design, much more consumed MZIs produce more phase-error and thermal crosstalk, resulting in apparent practical nonideal [5]. Additionally, we demonstrate the recognition of realistic hand-written digits captured by the camera (Figure 6f), further verifying the robustness of the PNN based on pseudo-real-value nonuniversal mesh when running in realistic world.

**Figure 5:**
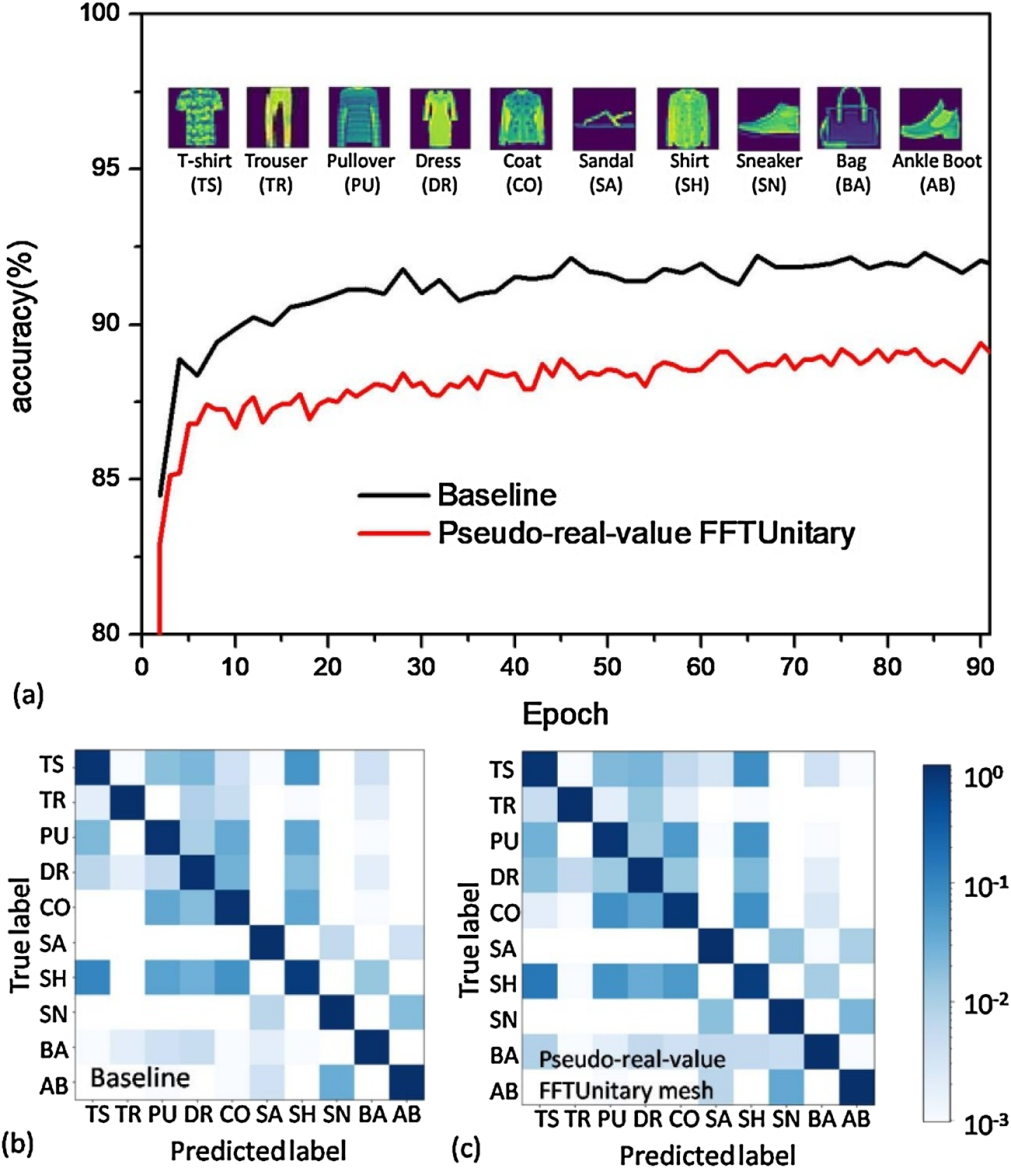
The performances of PNN on MobileNet model for Fashion-MNIST task using pseudo-real-value FFTUnitary mesh, as well as their comparision to the baseline uisng the reuglar universal real-value mesh. (a) The evolution of the Fashion-MNIST accuracy of MobileNet model; Confusion matrixes of (a) baseline prediction and (b) pseudo-real-value FFTUnitary mesh.

**Figure 6:**
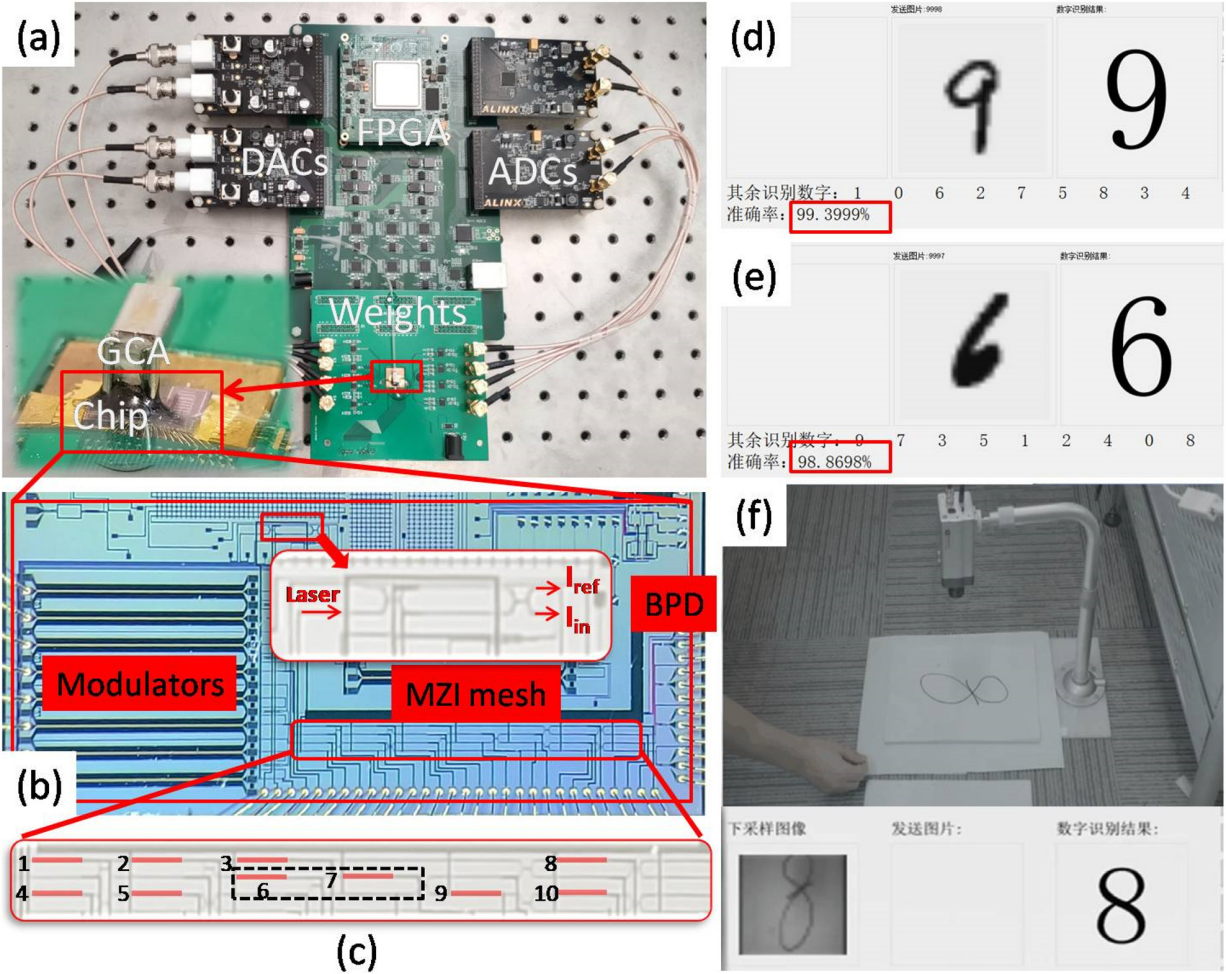
The system implementation of the PNN using pseudo-real-value MZI mesh. (a) PNN inferences system based on LeNet-5 network implemented on (b) silicon photonics chip with pseudo-real-value nonuniversal MZI mesh design, where (c) a P-GridUnitary mesh where 10 thermal shifters is employed to program five MZIs within (shifter 1 and 2 are *θ* and *ϕ* for 1st MZI, likewise, 4 and 5 for 2nd, 6 and 7 for 3rd, 3 and 8 for 4th, 9 and 10 for 5th). Tested MNIST accuracy when the chip works as (d) P-GridUnitary mesh or (e) FFTUnitary mesh. The demonstration of recognition of realistic hand-written digits captured by the camera.

**Figure 7:**
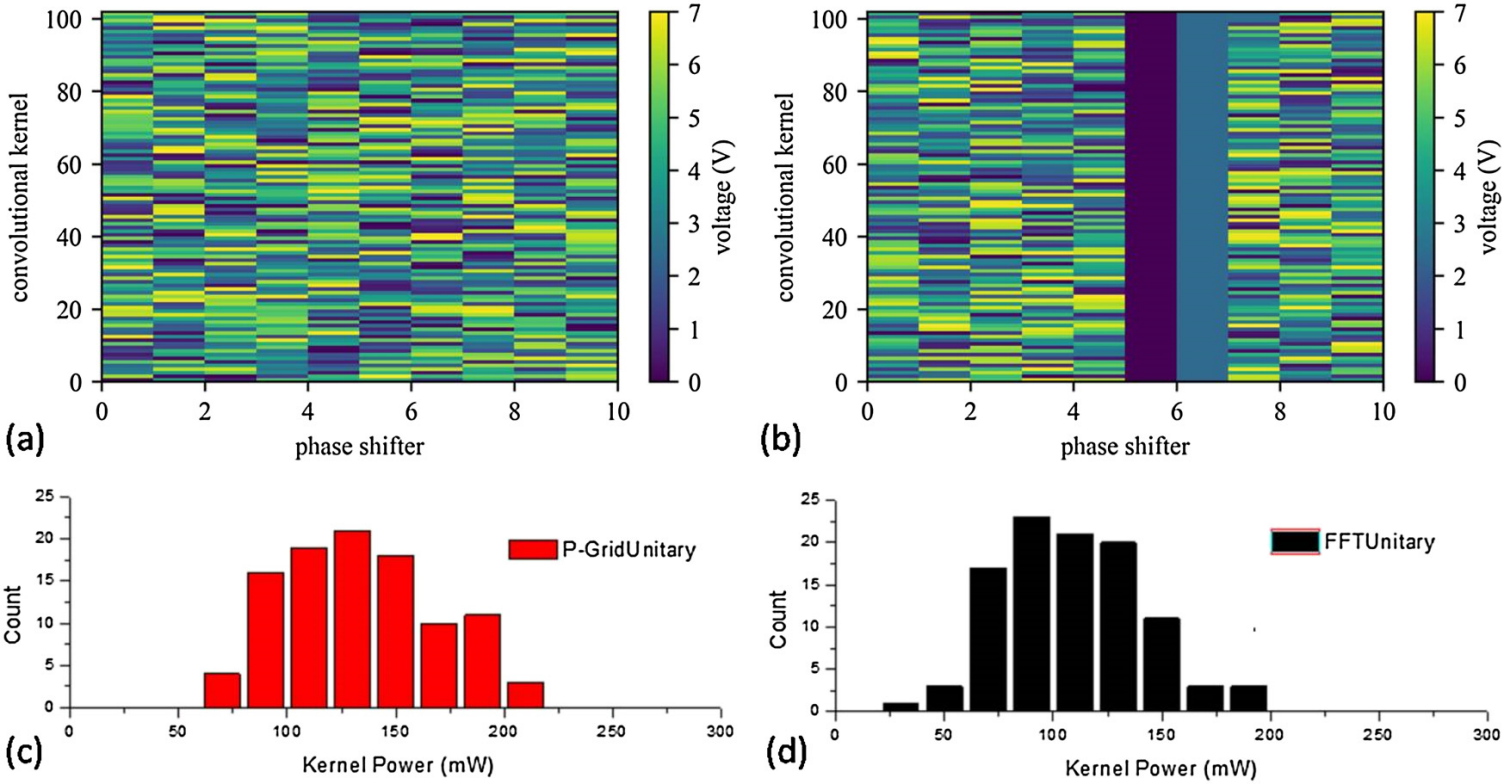
Voltage setting to configure the 102 kernels in the two convolutional layer of LeNet-5 model based on (a) pseudo-real-value P-GridUnitary mesh and (b) FFTUnitary mesh. Power consumed during configuring the kernel by pseudo-real-value (a) P-GridUnitary mesh and (b) FFTUnitary mesh.

**Figure 8:**
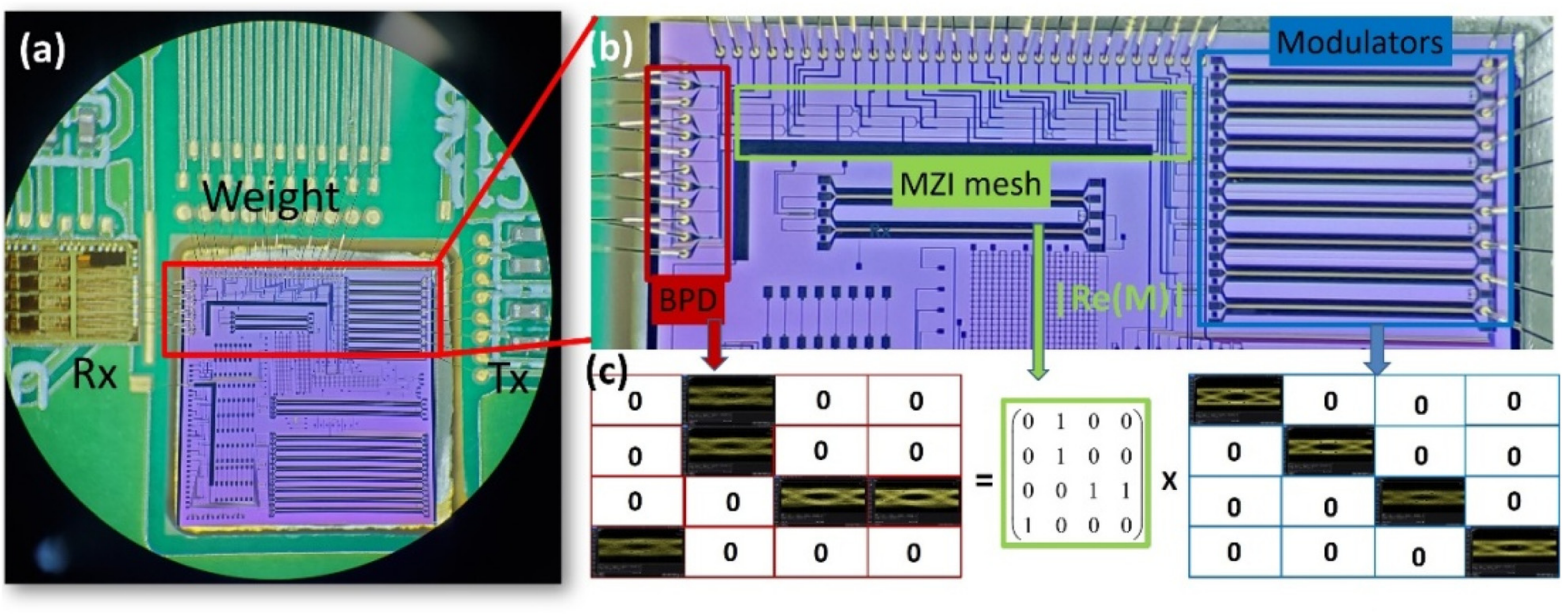
The feasibility of the pseudo-real-value MZI mesh to run matrix multiplication at high time-clock. (a) Packaged active silicon photonic chip with pseudo-real-value matrix multiplier; (b) Microscopy of the chip highlight the modulators array, balanced photon detectors (BPD) and MZI mesh; (c) matrix multiplication at 25 GHz demonstrated via eye diagram test.

During the inferences, the voltage values used to configure the 102 kernels in the two convolutional layer of our train LeNet-5 model based on pseudo-real-value P-GridUnitary mesh and FFTUnitary mesh is shown in Figure 7a and b, respectively. We combine the applied voltage with the resistance values (∼1.25 kΩ, SI-5) of the thermal shifter to calculate the power needed to configure all kernels. Hence, statistically, the averaged power consumption for the matrix programming is 134 mW for P-GridUnitary mesh and 108 mW for FFTUnitary mesh (if not accounting the power of the phase shifter to fix the marked MZI at cross state) as shown in Figure 7c and d). To further evaluate the potential of our pseudo-real-value mesh for high speed processing, as shown in Figure 8a–c, we demonstrate the 4 × 4 pseudo-real mesh working as the photonic matrix multiplier at high time clock frequency of 25 GHz, corresponding to the computation capability of 0.8TOPS. Considering the obtained averaged matrix programming power (note it is independent on the time clock frequency), as well as the typical power of the driver chip (Tx ∼ 1030 mW), the TIA chip (Rx ∼ 130 mW/Channel) and the laser (∼9 mW), the experimental energy efficiency is ∼0.48 TOPS/W. Furthermore, noting the possible low limit of the electrical power on modulator, photodetector and laser could be less than 1 mW/Channel [30], as well as the typical energy efficiency of digital–analog-convertor (DAC) and analog–digital-convertor (ADC) for photonic computing at ∼5.5 mW/Gsps [44], the potential energy efficiency of pseudo-real-value PNN with nonuniversal FFTUnitary mesh design is promising to reach ∼1.3 TOPS/W or better, even using the energy-inefficient thermal phase shifter as the weight element.

## 5 The scalability of the pseudo-real-value PNN

**Figure 9:**
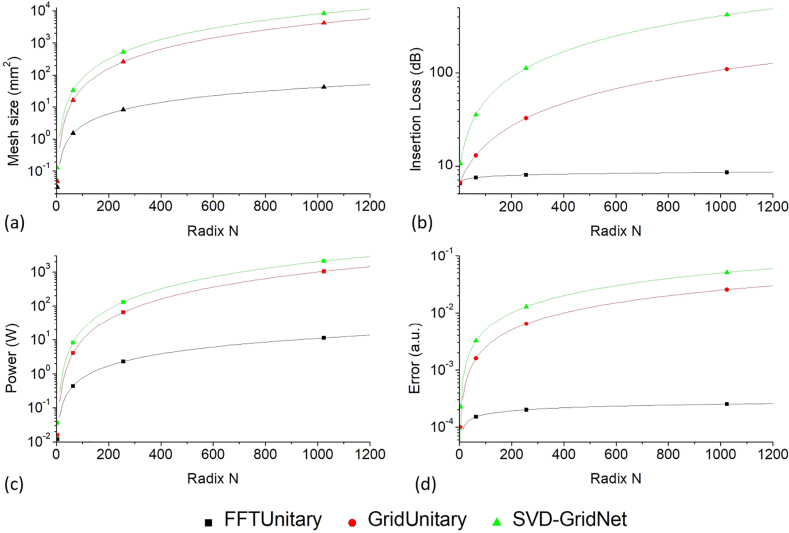
Comparison of the (a) mesh size, (b) insertion loss, (c) power consumption, (d) encoding error, among the pseudo-real-value nonuniversal FFTUnitary mesh (black) and universal GridUnitary mesh (red) as well as conventional SVD-GridNet mesh (green) as the functions of the radix *N*.

As shown in aforementioned sections, it is promising of our pseudo-real-value nonuniversal unitary mesh with MZIs least at *O*(N*log_2_ *N*) levels for PNN applications. In some particular tasks, including LeNet-5 for MNIST dataset, and MobileNet for Fashion-MNIST dataset, a most compact design using 0.5N*log_2_ *N* MZIs, pseudo-real-value FFTUnitary meshes, with either small or large radix *N* shows acceptable low classification accuracy loss. Also, we experimentally implement an inference system based on 4*4 pseudo-real-value nonuniversal unitary MZI mesh to show the feasibility of this architecture. Hence it is straightforward to substitute the conventional SVD MZI mesh with pseudo-real FFTUnitary mesh as the building block for weight matrix multipliers in the applications like PNN, then the computational power and chip area needed for mesh-construction as well as the calibration complexity are reduced remarkably, scaling with the radix of the matrix *N* as *N*log_2_ *N*/2. As such, highly differing from the conventional electrical matrix multiplier which upgrades the performances *via* scaling-down of the transistor size and are facing performance-limitation due to the ending of Moore’s Law, the improvement of the photonic matrix multiplier for ANN applications can be beneficial from the scaling-up of the dimension *N* of the matrix represented by the pseudo-real nonuniversal unitary MZI mesh. Especially, there could be a rather high improvement space since the size of a die could be far larger than that of an MZI cell, allowing ultra large scale, e.g., pseudo-real FFTUnitary mesh. The scale laws of the chip size, optical loss, programming power and encoding error for PNN based on SVD-based GridNet mesh, pseudo-real-value universal pseudo-real GridUnitary mesh and nonuniversal pseudo-real FFTUnitary mesh are listed in Table 1 (SI-7). Quantitatively, the performances of these three kinds of mesh are evaluated and compared in Figure 9a–d assuming using state of art photonic components [17, 30, 45], [46], [47] (SI-7). It clearly present the all-round advantages of pseudo-real FFTUnitary mesh especially when the radix *N* increases. However, for the case of small radix *N*, the intrinsic large size of MZI (∼8000 μm^2^) make even the pseudo-real FFTUnitary mesh area-inefficient comparing to the photonics matrix multiplier based micro ring resonator (∼250 mm^2^) [29]. This implies that it is still quite significant to the optimize the photonic device, e.g., shrinking the size of MZI *via* optimized design as well as introducing the silicon-photonics-process compatible but more efficient phase shifter [48], besides the exploration on architecture-improvement. They co-open the room to upgrade the performances of the photonic chip for neural network applications.

**Table 1:** Performance comparison among SVD-based GridNet mesh, pseudo-real GridUnitary mesh and pseudo-real FFTUnitary mesh for PNN applications.

Mesh type	SVD-based GridNet mesh	Pseudo-real GridUnitary mesh	Pseudo-real FFTUnitary mesh
Mesh size	*N* ^2^ *S*	*N*(*N* − 1)*S*/2	*N*(log_2_ *N*)*S*/2
Optical loss	O(2*N* + 1)	O(*N*/2)	*O*(log_2_ *N*/2)
Programming power	∼2*N* ^2^ *P_π_*	∼*N*(*N* − 1)*P_π_*	∼*N*(log_2_ *N*)*P_π_*
Encoding error	*O*(2*N* + 1)	*O*(*N*)	*O*(log_2_ *N*/2)

## 6 Conclusions

In conclusion, we propose the pseudo-real mesh to implement scalable and compact PNN, which utilizes the real part of the complex-value matrix defined by a nonuniversal unitary MZI mesh to express the real-value matrix. Qualitatively, this method allows nonuniversal unitary mesh consuming *O*(*N*log_2_ *N*) level MZIs to achieve high accurate matrix expressivity close to conventional universal SVD mesh require O(*N* ^2^) MZIs at small radix *N*, while at large radix limit, it is also promising for the pseudo-real mesh with *O*(*N*log_2_ *N*) MZIs to approach the weight kernels for PNN applications due to their low-dimension embedding tendency. We train the LeNet-5 model for MNIST dataset and MobileNet model for Fashion-MNIST dataset, verifying the compact pseudo-real-value FFTUnitary mesh design could apparent reduce the required photonic devices but pay acceptable low cost on learning capability loss. Experimentally, we implement an inference system based on 4*4 pseudo-real-value photonic neural chip, which run the LeNet-5 network for MNIST dataset with high accuracy to demonstrate the feasibility of our photonic computation architecture. On the view point of technical implementability, the pseudo-real mesh design shows all-round advantages over conventional SVD-based mesh design on chip size, insertion loss, power consumption and encoding error. Further improvement may be achieved by shrinking the size of MZI *via* optimized design as well as introducing the silicon-photonics-process compatible but more efficient phase shifter. Our results, presented in this paper, may inspire some further interdisciplinary explorations teaming up with the researchers in computer science and mathematics community on the exact potential of pseudo-real-value nonuniversal unitary mesh for scalable photonic processing.

## Supplementary Material

Supplementary Material
