# Supplementary material for: Scalable and compact photonic neural chip with low learning-capability-loss
Source: Nanophotonics. 2021 Dec 22;11(2):329–44. doi: 10.1515/nanoph-2021-0521 (PMC11501640; doi:10.1515/nanoph-2021-0521)
Supplement: Supplementary file 3 — Supplementary Material [file j_nanoph-2021-0521_suppl.docx]

## Supporting Information

Scalable photonic neural chip with low learning-capability-loss

Ye Tian, Yang Zhao, Shengping Liu, Qiang Li, Wei Wang, Junbo Feng, Jin Guo

^1^ Chongqing United Microelectronics Center (CUMEC), Chongqing 401332, China

[*junbo.feng@cumec.cn](mailto:*junbo.feng@cumec.cn)

1 The treatment to non-zero and non-uniform of the phases of the reference source

In the mainbody of the paper, we assume the initial phase of the reference sources as 0. However, due to the fabrication imperfection, initial phases of the reference sources for interference with the output signal from different ports are almost impossible to ideal 0. Their value may be non-zero and non-uniform (not equal to each other). However, such non-zero and non-uniform initial phases won’t affect the availability of pseudo-real mesh architecture. Actually, the interference of output (the phase is *φ_out_*) with reference source with non-zero *φ_ref_* in eq.4 and/or eq.5 can be deemed as a “virtual” output whose phase is (*φ_out_*-*φ_ref_*) interference with reference source with zero initial phase. The “virtual” output *E_virtual-out_* is correlated with the realistic output with a diagonal matrix *D* determined by the reference source phases

(S1)

Hence we modify the eq.3 in the mainbody as

$\mathrm{Re}(E_{virtual-out})=\mathrm{Re}(DE_{out})=\mathrm{Re}(DUE_{in})=\mathrm{Re}(DU)E_{in}$ (S2)

Such that, *E_virtual-out_* can be deemed as *E_out_* in eq.3, and likewise *DU* can be deemed as *U*. Then the algorithm used for pseudo-real mesh would still work well. e.g., the numerical study to the influences of the non-zero and non-uniform phase to the unitary matrix expressivity of the 4*4 FFTUnitary mesh is shown in Fig. S1, little influences are observed.


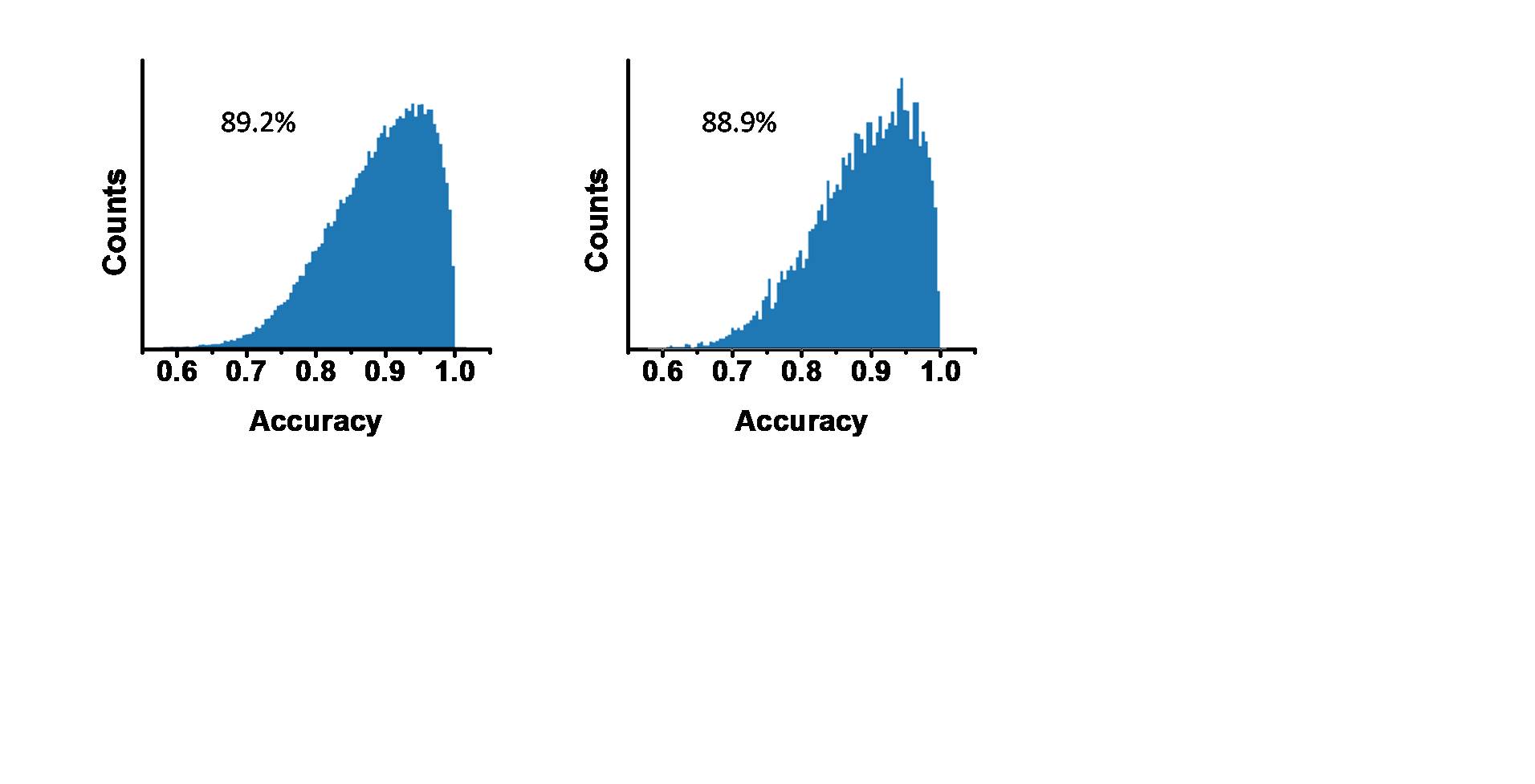


Fig. S1 The statistical accuracy distribution of the unitary matrix expressed by the 4*4 FFTUnitary mesh (a) without or (b) with non-zero and non-uniform reference phases.

2 The numerical approaching of pseudo-real non-universal mesh to target real-value matrix

Without the loss of the generality, here we take the case of pseudo-real FFTUnitary mesh as the instance to propose the details to approach real-value matrix using pseudo-real non-universal unitary mesh. Exactly, we use the real part of the S-matrix of pseudo-real FFTUnitary mesh to fit the arbitrary real-value matrix. However, practically, the given real-value matrix often cannot fulfill the condition of *β*=1 and all singular values are less than 1, hence here we present a more feasible algorithm flow to implement arbitrary real-value matrix by pseudo-real 4x4 FFTUnitary mesh:

The first step is to find the unitary matrixes, whose real part is equal to the arbitrary real-value matrix. Assuming the arbitrary real-value matrix is *M*, we apply SVD (Singular Value Decomposition) to *M*:

$\begin{matrix} M\text{=}U\Sigma V^{H} \\ \text{where} \Sigma=\left[\begin{matrix} \sigma_{1} & 0 & 0 & 0 \\ 0 & \sigma_{2} & 0 & 0 \\ 0 & 0 & \sigma_{3} & 0 \\ 0 & 0 & 0 & \sigma_{4} \end{matrix}\right] \end{matrix}$ (S3)

Where *U* and *V^H^* are unitary matrixes, and Σ is a diagonal matrix. The values in the diagonal of Σ is the singular values of *M*. If the max singular value is larger than 1, *M* cannot be realized by MZI mesh. Thus we divide all the singular values by the max singular value, and *M* becomes:

$\begin{matrix} U\Sigma_{1}V^{H}=\frac{M}{\max(\sigma)} \\ \text{where} \Sigma_{1}=\frac{\Sigma}{\max(\sigma)}=\left[\begin{matrix} \sigma_{11} & 0 & 0 & 0 \\ 0 & \sigma_{12} & 0 & 0 \\ 0 & 0 & \sigma_{13} & 0 \\ 0 & 0 & 0 & \sigma_{14} \end{matrix}\right] \\ \text{as} \max(\sigma)=\max(\sigma_{1}, \sigma_{2}, \sigma_{3}, \sigma_{4}) \end{matrix}$ (S4)

Then we find the imaginary part of the unitary matrixes. We find the diagonal matrixes Σ_2_, who’s each element on the unitary satisfy σ_1i_^2^+ σ_2i_^2^=1. Therefore, Σ_u_=Σ_1_+iΣ_2_ satisfy Σ_u_Σ_u_*^H^*= Σ_u_*^H^*Σ_u_ =*I*, which means Σ_u_ is a unitary matrix. As *U*, Σ_u_, *V^H^* are all unitary matrix and the matrix multiplication product *U*Σ_u_*V^H^* is also unitary matrix. So the unitary matrix *U*Σ_u_*V^H^* can be realized by a photonic network, and the real part of S-matrix of the photonic network is actually $\frac{M}{\max(\sigma)}$. Specified,

$\begin{matrix} U\Sigma_{u}V^{H}=U(\Sigma_{1}+i\Sigma_{2})V^{H}=U\Sigma_{1}V^{H}+iU\Sigma_{2}V^{H} \\ \mathrm{Re}\{U\Sigma_{u}V^{H}\}=U\Sigma_{1}V^{H}=\frac{M}{\max(\Sigma)} \\ \text{where} \Sigma_{2}=\left[\begin{matrix} \pm\sigma_{21} & 0 & 0 & 0 \\ 0 & \pm\sigma_{22} & 0 & 0 \\ 0 & 0 & \pm\sigma_{23} & 0 \\ 0 & 0 & 0 & \pm\sigma_{24} \end{matrix}\right] \\ \text{as} \sigma_{1i}^{2}+\sigma_{2\text{i}}^{2}=1 \\ \Sigma_{u}=\Sigma_{1}+i\Sigma_{2}\text{=}\left[\begin{matrix} \sigma_{11}\pm i\sigma_{21} & 0 & 0 & 0 \\ 0 & \sigma_{12}\pm i\sigma_{22} & 0 & 0 \\ 0 & 0 & \sigma_{13}\pm i\sigma_{23} & 0 \\ 0 & 0 & 0 & \sigma_{14}\pm i\sigma_{24} \end{matrix}\right] \\ \text{as} \Sigma_{u}\Sigma_{u}^{H}=\Sigma_{u}^{H}\Sigma_{u}=I, \Sigma_{u} \text{is} \text{unitary} \text{matrixes} \\ U, \Sigma_{u}, V^{H} \text{are} \text{all} \text{unitary} \text{matrixes, so} U\Sigma_{u}V^{H} \text{is} \text{unitary} \text{matrixes} \end{matrix}$ (S5)

The second step is to find how to realize the unitary matrix *U*Σ_u_*V^H^* by pseudo-real FFTUnitary mesh. If we use rectangular mesh like Clements did, this step can be done by matrix decomposition. However, there is still no matrix decomposition method for pseudo-real FFTUnitary mesh. Some previous works shows that the numerical approaching of FFTUnitary mesh to random Hessian matrixes with dimension 64*64 by stochastic gradient descent (SGD) backward propagation (BP) seems not accurate enough, and more powerful optimization methods is desired to approach the theoretically expected matrix expressivity [[1](#_ENREF_1)]. Moreover, the training time would grow exponentially since we need to do 2^N^ times training to approach the FFTUnitary mesh to all available unitary matrixes and range their approaching error to pick out the minimal-error one. Even though, the BP-based numerical experiments for the expressivity of pseudo-real FFTUnitary mesh design with small matrix dimension like 4*4 is still feasible and provide us the clues about its nearly equivalent expressivity to that of regular SVD-based mesh.

For the case of approaching 4*4 matrix, firstly, we generate 10,000 random arbitrary target real matrices, and use the method from step 1 to find its corresponding unitary matrices. As each real matrix has 2^4^ = 16 kinds of unitary matrices when dimension is equal to 4, now we get 160,000 target unitary matrices.

Next, we choose 160,000 sets of tunable phase values on the MZIs of the 4x4 pseudo-real FFTUnitary mesh as the input of the BP algorithm. We also construct the S-matrix calculation function of the FFTUnitary mesh, which is the forward propagation process of the BP algorithm, and the output of the BP algorithm is 160,000 sets of the S-matrices of the 4x4 pseudo-real FFTUnitary mesh.

Finally, we use Tr [Re(Y*D^H^)] to evaluate the similarity between the output S-matrices Y and the target unitary matrices D, and use BP algorithm to find the optimal input to get the best accuracy. After training, we learn that the average accuracy of 4x4 pseudo-real FFTUnitary mesh is around 89.1%. Furthermore, as we only need to find one S-matrix whose real part is equal to the target real matrices, we can choose the best S-matrices from the 16 corresponding output S-matrices. By this method, we can boost the average accuracy up to 97.1% as the Hist diagram shown in Figure S1. This is very close to the theoretical expected 98.4% (note error is ~1/(2^4^*4)=1.6%). Using the similar flow, we can learn the matrix (both unitary and real-valued) by pseudo-real non-universal P-GridUnitary mesh and universal GridUnitary mesh.

Likewise, the cases for pseudo-real 8*8 FFTUnitary mesh and 8*8 Stacked-FFTUnitary mesh are studied, but considering the efficiency of the algorithm, only 100 random real-value matrixes are learned, which typically take ~1days. While for the 16*16 case, since the picking times are 2^8^ higher than the case of 8*8, and learning single 16*16 unitary matrix is also more time-consuming, it is quite challenge for our computer to obtain statistical-meaningful results. And we wish to team-up with some other CS team with GPU card to make further explorations.


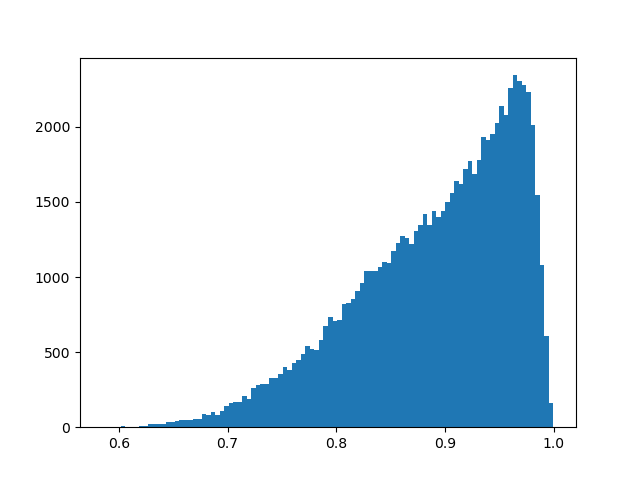

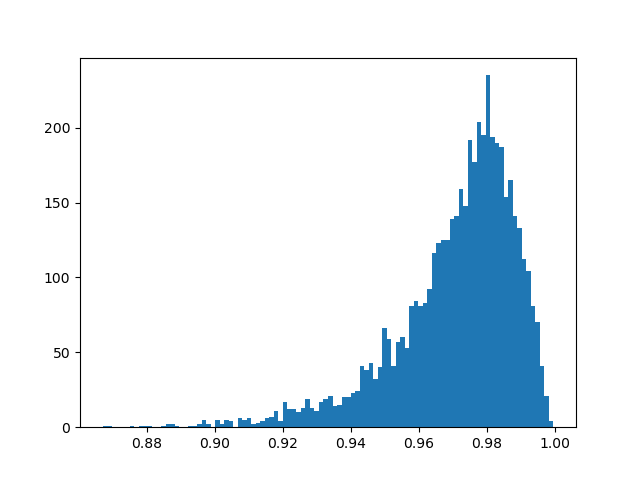


Figure S2. Left is the Hist diagram of the accuracy between the target unitary matrices and each corresponding output S-matrices. Right is the Hist diagram of the accuracy between the target real-value matrices and its corresponding output S-matrices with highest accuracy

*3 The estimation to the mathematic expectation of the error for given distribution.*

Let’s consider the error follows the distribution f(x), then we sample the f(x) for 2^N^ times, and write the minima-error Y of 2^N^ samples $(X_{1},X_{2}......X_{2^{N}})$as $Y=\min(X_{1},X_{2}......X_{2^{N}})$. If assume all the samples X_i_ are mutual independent, then the probability of Y<E should be

$\begin{matrix} F_{Y}(E)=P(\min(X_{1},X_{2}......X_{2^{N}})<E) \\ =1-P(\min(X_{1},X_{2}......X_{2^{N}})>E) \\ =1-P_{1}(X_{1}>E)P_{2}(X_{2}>E)......P_{2^{N}}(X_{2^{N}}>E) \\ =1-[P_{1}(X_{1}>E)]^{2^{N}} \\ =1-[1-\int_{-\infty}^{E} f(x)dx]^{2^{N}} \end{matrix}$ (S6)

Then the probability density function of Y would be

$f_{Y}(E)=\frac{dF_{Y}(E)}{dE}=2^{N}[1-\int_{-\infty}^{E} f(x)dx]^{2^{N}-1}f(E)$ (S7)

Hence the mathematic expectation of Y is

$\int_{-\infty}^{+\infty} Ef_{Y}(E)dE=\int_{-\infty}^{+\infty} 2^{N}[1-\int_{-\infty}^{E} f(x)dx]^{2^{N}-1}f(E)EdE$ (S8)

Considering the range of the fidelity-dependent error is in [0,1], Eq.S can be re-written as

$\int_{0}^{1} Ef_{Y}(E)dE=\int_{0}^{1} 2^{N}[1-\int_{0}^{E} f(x)dx]^{2^{N}-1}f(E)EdE$ (S9)

This is an integral-form description to the mathematic expectation of the error. Given that we can obtain the analytical form of the fidelity distribution, its exact value can be calculated. While using assumed Gaussian or uniform distribution, it is possible to restrict the available range of the mathematic expectation.

*4 Further discussions to the relationship between the matrix expressivity and the required MZIs of pseudo-real-value mesh*

As shown in Fig. S3, current record on the computation complexity of the dense matrix multiplication is O(N^2.3728596^), indicating that the dense matrix is probable approximated by mesh using O(N^1.3728596^) or more MZIs. Moreover, Ran Raz proved a lower-bound to the computational complexity of the matrix multiplication as O(N^2^logN) [[2](#_ENREF_2)] but this bound is only available for approaching the sparse matrix: Likhosherstov, et.al., from Google theoretically proved based on the Johnson-Lindenstrauss lemma[[3](#_ENREF_3)], and experimental verified the log-dimension embedding for the expressive power of (sparse) self-attention matrices [[4](#_ENREF_4)] Given this, it is expected that the mesh with reduced O(NlogN) MZIs could perform well on DNN applications considering the machine learning feature tend to low-dimension. Additionally, from the log-log plot to the computation complexity (Fig. S3), it founds that O(N^2^logN) grow slowly than O(N^2.3728596^) when the radix N become large, in line with the fact that the algorithm of matrix multiplication with O(N^2^logN) computation complexity is more challenge than the counterpart with O(N^2.3728596^) computation complexity. While for small N, the effective power number of N^2^logN is bigger than 2.3728596 (see the N^2^Log_2_N line is higher than N^2.3728596^ line in small N range). As such, it is admitted to approximate arbitrary dense matrix accurately with O(NlogN) MZI mesh as shown in the mainbody.


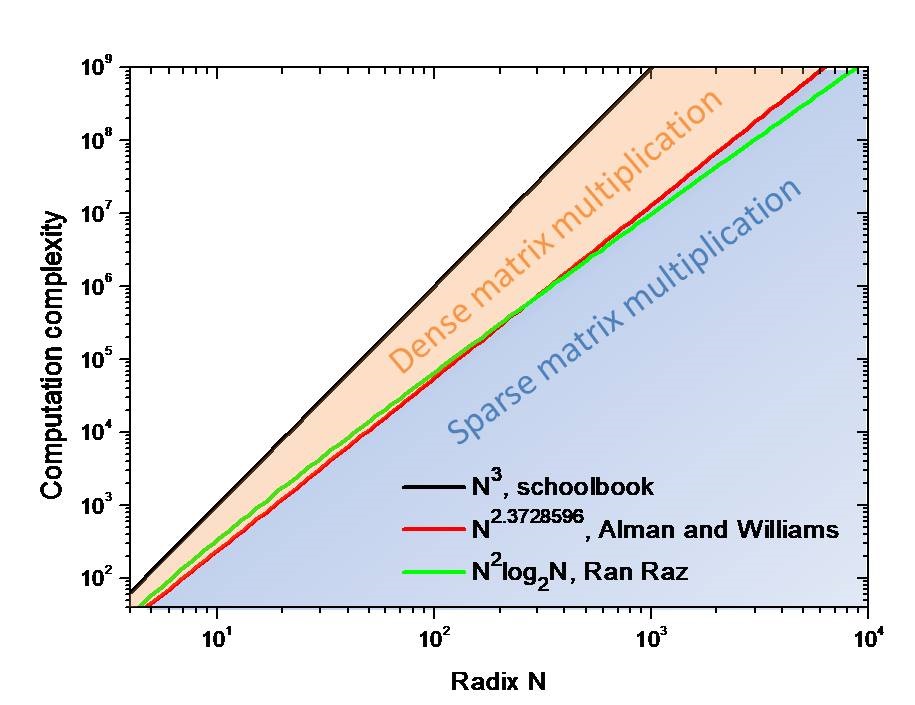


Fig. S3 the bound of computation complexity for arbitrary dense and/or sparse matrix multiplication (According to current algorithms, arbitrary dense matrix multiplication is only possible with O(N^2.3728596^) or high computation complexity, accordingly, sub-O(N^2.3728596^) is only feasible for sparse matrix multiplication)

5 LeNet-5 network for MNIST task and MoblieNet for Fashion-MNIST


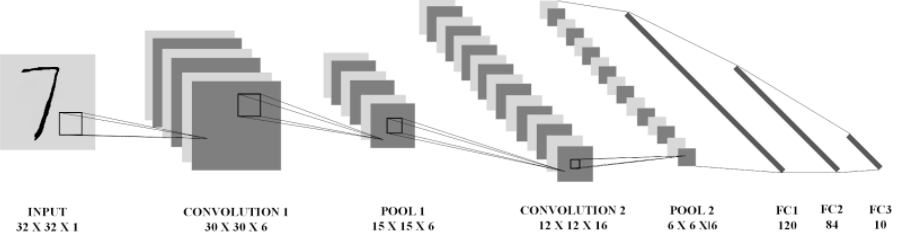


Fig. S4 Schematic illustration of the LeNet-5 networks

Fig. S4 schematically illustrate the LeNet-5 networks for hand-write-digits recognitions (MNIST dataset). The dataflow to implement LeNet-5 network on our system for MNIST inference as well as its model training are as following:

5.1 data pre-processing

In this task, we add zero padding in each sides of the picture of MNIST task to make the picture size from 28x28 to 32x32 to make the data more similar to the actual case. And we shuffle the 60,000 training set and the 10,000 test set of the MNIST to increase the randomness.

5.2 Forward Propagation

The neural network of our LeNet-5 network consists of 5 layers:

The first layer is a 6-out channel 3x3 convolution layer with a 2x2 Max pooling layer and a Relu activation function. Data go through this layer from 32*32 image to 6 15*15 images

The second layer is a 16 out channel 6 in channel 4x4 convolution layer with a 2x2 Max pooling layer and a Relu activation function. Data go through this layer from 6 channel 15*15 image to 16 channel 6*6 images. Then we reshape the 16 6*6 images to a vector with 576x1size.

The third layer is a 576x120 fully connected layer with a Relu activation function. Data go through this layer from 576x1vector to 120x1 vector.

The forth layer is a 120x84 fully connected layer with a Relu activation function. Data go through this layer from 120x1 vector to 84x1 vector.

The fifth layer is an84x10 fully connected layer with a Softmax activation function. Data go through this layer from 84x1 vector to 10x1 vector.

Among all the network, we run all the MAC (Multiply Accumulate) computation of the convolution layer on photonic chip, and run other computation on FPGA.

To compensate the non-linear correlation between the amplitude detection and intensity modulation, E∝I^0.5^∝U^0.5^ , where U is output voltage, I and E is light intensity and real part of input field after modulation respectively. we insert a square-root operation in two convolutional layers to reflect the influences of such conversion

5.3 Backward Propagation

We use the auto-grad function of Pytorch to do backward propagation. In the example, the loss function is defined by the cross entropy between the forward propagation results and the pre-labeled classes of MNIST Datasets.

5.4 Architecture of MobileNet

The architecture of MobileNet is shown in Table S1. It starts with a 3x3 Pointwise convolution layer with batch norm and ReLU like Fig. 1 Left), which increases the feature map channel to 32 and halve the feature map size with stride 2. The next 13 layers are Depthwise Separable convolutions consisting of 3x3 Depthwise and 1x1 Pointwise convolution layers followed by batch norm and ReLU like Fig. 1 Right).Normally, the Depthwise convolutions handle down sampling while the Pointwise convolutions expand channel. Finally, an average pooling reduces the feature map size to 1, and a fully connected layer followed by a SoftMax layer adjusts the feature map channel to number of datasets classes.

Table S1. MobileNet Architecture

| Type / in channel / out channel / stride | Input Size（Origin） | Input Size（Ours） |
| --- | --- | --- |
| Conv3x3 / ic3 / oc32 / s2 | 224 x 224 x 3 | 56 x 56 x 3 |
| DSConv / ic32 / oc64 / s1 | 112 x 112 x 32 | 28 x 28 x 32 |
| DSConv / ic64 / oc128 / s2 | 112 x 112 x 64 | 28 x 28 x 64 |
| DSConv / ic128 / oc128 / s1 | 56 x 56 x 128 | 14 x 14 x 128 |
| DSConv / ic128 / oc256 / s2 | 56 x 56 x 128 | 14 x 14 x 128 |
| DSConv / ic256 / oc256 / s1 | 28 x 28 x 256 | 7 x 7 x 256 |
| DSConv / ic256 / oc512 / s2 | 28 x 28 x 256 | 7 x 7 x 256 |
| 5 x DSConv / ic512/ oc512 / s1 | 14 x 14 x 512 | 4 x 4 x 512 |
| DSConv / ic512 / oc1024 / s2 | 14 x 14 x 512 | 4 x 4 x 512 |
| DSConv / ic1024 / oc1024 / s1 | 7 x 7 x 1024 | 2 x 2 x 1024 |
| Avg Pool / ic1024 / oc1024 / s1 | 7 x 7 x 1024 | 2 x 2 x 1024 |
| FC / ic1024 / oc1000 (ours: 10) / s1 | 1 x 1 x 1024 | 1 x 1 x 1024 |
| SoftMax / ic1000 (ours: 10)/ oc1000 (ours: 10) / s1 | 1 x 1 x 1000 | 1 x 1 x 10 |

The input size of MobileNet and Fashion MNIST is 224x224x3 and 28x28x1. So, we resize the images in Fashion MNIST to 56x56x3 to reduce feature loss, which is a tradeoff between feature loss and training time. The input size of each layers is shown in Table 1. As we us FFT unitary chip to run the MobileNet, we use the real part of the S matrix of FFT unitary chip to replace the weight of convolution layers. For example, we use the first 3 rows and 3 columns of the real part of S matrix of 3x32 pieces of 4x4 FFT unitary mesh to replace the weight of 3x3x3x32 convolution layer, and use the first 32 rows of the real part of S matrix of 1 piece of 64x64 FFT unitary mesh to replace the weight of 1x1x32x64 convolution layer. Likewise the weight of 1x1x64x128 convolution layer is represented by 128x128 FFT unitary mesh, etc. We use Adam optimizer to train 90 epochs, and the learning rate is 0.001.

Fig. S5. Left: 3x3 Pointwise convolution layer with batchnorm and ReLU. Right: Depthwise Separable convolutions consisting of 3x3 Depthwise and 1x1 Pointwise convolution layers followed by batchnorm and ReLU

6 Phase shifter characterization

The MZIs are modulated by thermo-optic effect. The thermal is provided by the integrated heater. The designed waveguide is with cross section of 450 × 220 nm^2^ . The TiN heater has length of 150 μm, width of 2 μm and thickness of 120 nm. The measured resistances of all heaters are around 1.25kΩ (The resistance values of all 10 heaters are listed in Table S1). We apply the voltage sweeping (0-7V, 5mV interval, maintain 200μs on each step) on the heater one-by-one and observe the signal y received by conresponding ADC channels, which is proportional to the photocurrent with the coefficient given by the transimpedance of TIA (transimpedance amplifier). Since the photocurrent is determined by the light power output from the mesh, we can annalistically describe the received signal y as the function of the voltage applied on the heater [[5](#_ENREF_5)]:

*y=a*cos(b*V^2^/R +c)+d* (S10)

where *d* was a constant background, *a* was the maximum magnitude of the signal, *b* and *c* were coefficients depicting the relationship between the phase and the electrical voltage*.* We show the test results of several exemplary heaters in Fig. S6. The fitting to the measurements always exhibit 𝑅^2^ -values high than 0.99 (the best to reach is 1), indicating that the model adequately reproduces the data observed from the measurements. Hence we can extract the shifting efficiency of corresponding heater as Pπ=π/b. The shifter efficiency of all 10 heaters are listed in Table S2, and the averaged efficiency is ~21mW.

Table S2 The resistance value and shifter efficiency of ten heaters for thermal programming of MZI mesh

| No. | 1 | 2 | 3 | 4 | 5 | 6 | 7 | 8 | 9 | 10 |
| --- | --- | --- | --- | --- | --- | --- | --- | --- | --- | --- |
| R /kΩ | 1.244 | 1.250 | 1.244 | 1.252 | 1.252 | 1.246 | 1.254 | 1.249 | 1.250 | 1.252 |
| Pπ/mW | 21.26 | 20.19 | 22.07 | 19.78 | 22.90 | 20.81 | 19.83 | 20.54 | 20.98 | 22.09 |

Fig. S6 Fitting curve of the calibration of several exemplary phase shifters


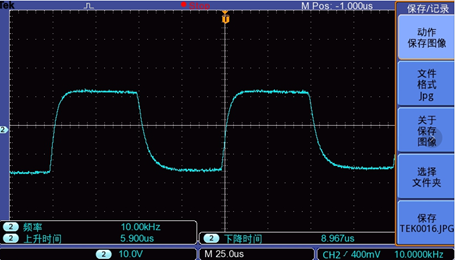


Fig. S7 temporal response of the heater for MZI programming

The typical rising time and fall time of the MZI cell is 5.9 μs and 8.97 μs (Fig.S7), much shorter than the duration maintained in each step (200 μs). This ensure the aforementioned test could get reliable results after all the heater is in the stable state.

7 The matrix learning performed on inference system

The trained LeNet-5 model has 96 4*4 and 6 3*3 kernels. Once their corresponding phase values are correctly programmed on the chip, then the real part of the mesh would be the obtained real-value matrix. Considering the operation flow of our pseudo-real-value mesh is something like the real encoding & real detection to the complex-valued neural networks, here we calibrate the mesh using the method similar to that done in [[5](#_ENREF_5)], and then executing the matrix programming. e.g., for matrix expressed using 4*4 pseudo real-value FFTUnitary mesh, the typical error is about ~5%.

*
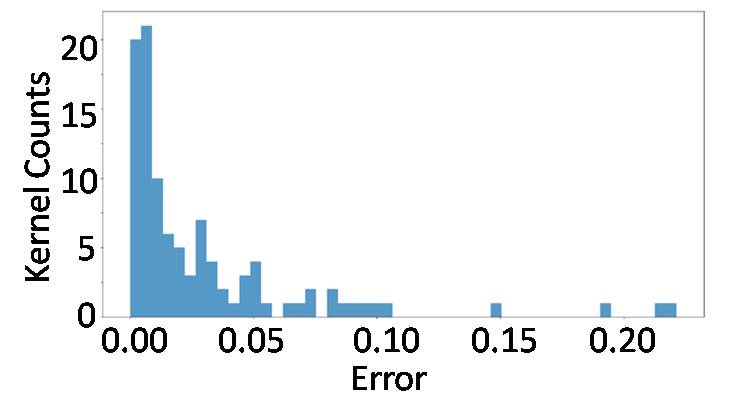
*

Fig. S8 statistical error of real value matrixes expressed by 4*4 pseudo real-value FFTUnitary mesh, where the 102 kernels from LeNet-5 model is used as the instances for the study.

7 The detail of the evolution to the advantages of pseudo-real-value FFTUnitary mesh

**1) Chip size**: chip area of the mesh for N*N matrix is proportional to the number of MZI needed for construction, It is N^2^S for SVD-based GridNet mesh, N(N-1)S/2 for pseudo-real GridUnitary mesh and Nlog_2_NS/2 for pseudo-real FFTUnitary mesh, respectively (S is the size of a MZI cell). This size estimation seems relative rough because the area of the Y-shape splitter for 1xN splitting, the low-loss crossing in FFTUnitary mesh as well as the modulator and photo detector is not considered. However, the Y-shape splitter and crossing are much smaller than MZI cell or can be substituted by 3D layers waveguide[[6-8](#_ENREF_6)], while the modulators and photo detectors scale with O(N) to the matrix dimension N[[9](#_ENREF_9)]. Hence for the large N, ignoring their area would not affect the primary conclusion of our analysis. It founds pseudo-real FFTUnitary mesh requires 204.8x fewer MZIs at the radix of N=1024 compare to SVD-based GridNet mesh. Accordingly, the chip (mesh) size could be just ~41 mm^2^ if using the state of art MZI cell with footprint of ~8000 μm^2^ [[10](#_ENREF_10)], indicating the great potential of our architecture to highly scalable PNN.

**2) Optical loss:** As the input modes propagate through MZI mesh, the power carried by the output modes will unavoidable lower than that of the input modes due to the loss generated in the mesh. Hence the optical loss should be proportional to the optical depth of the mesh. It is 2N+1 for SVD-based GridNet mesh, N for pseudo-real GridUnitary mesh and log_2_N for pseudo-real FFTUnitary mesh, respectively. On the other hand, for the cases of pseudo-real mesh, part of the light source is divided and used for reference source which exactly do not pass through the mesh. So, the actual loss could further decrease to the same level of a “virtual” mesh with optical depth of N/2 for pseudo-real GridUnitary mesh and (log_2_N)/2 for pseudo-real FFTUnitary mesh if the light source is equally splitting for referencing and computing (i.e., μ=0.5). Actually, for the photonic computation chip with conventional SVD-mesh design, we can see that the light injected into the chip and then splitting into N input ports, they would pass thorough the modulators array, the mesh and then “seen” by the photon detector array. The loss would be (if not accounting the deliberately attenuation produced by the diagonal matrix to define different singular values)

$IL_{\text{SVD}}=IL_{Coupler}+IL_{Splitter}+IL_{Mod}+IL_{Mesh}$ (S11)

While for pseudo-real-value mesh, the light “seen” by the balanced photon detector is

(S12)

Let’s set μ=0.5 then $I=I_{+}-I_{-}=\text{=}\sqrt{I_{in}}\alpha ME_{in}$. This is something like that only half of all the input light power propagate through the modulator array and the MZI mesh, while the rest half part just propagate on low-lossy waveguides and does not suffer the attenuation produced by the modulator array and the MZI mesh. Such that we get the loss of the pseudo-real-value mesh as

$IL_{pseudo}=IL_{Coupler}+IL_{1\times2MMI}+IL_{Splitter}+\frac{IL_{Mod}+IL_{Mesh}}{2}+IL_{Mixer}$ (S13)

Specified, the coupler, the splitter, and the modulator (also the MMI and the mixer for pseudo-real-value mesh) contributes O(1) loss, while the IL of the mesh itself is proportional to the optical depth. If assume the loss of the MZI is ~0.2dB (e.g., contributed by two cascaded 50:50 directional coupler with typical loss of 0.1dB), the loss of the 3D cross used for MZI stage interconnection is 0.1dB[[11](#_ENREF_11)], IL_coupler_=3dB, IL_MMI(Mixer)_=0.1dB, IL_splitter_=0.1*log_2_N (we use the power splitter tree based on 1x2 MMI with typical loss of 0.1dB, which consume log_2_N stage to provide 1:N equal splitting), IL_Mod_=5.5dB, the loss of a pseudo-real-value FFTUnitary mesh for NxN matrix multiplier would be 6.45+0.5* (0.2+0.1+0.2) *log_2_N. Hence, assuming the insertion loss of the optical coupler, MMI and Modulator, MZI, and 3D crossing is 3dB, 0.1dB, 5.5dB, 0.2 and 0.1dB respectively[[6-8](#_ENREF_6), [12](#_ENREF_12), [13](#_ENREF_13)], the conventional SVD-based GridNet mesh produces an insertion loss of 419.5dB at the radix of N=1024, needing ultra high optical gain to compensate. Whereas the insertion loss of our pseudo-real FFTUnitary mesh would be only 8.45dB and quite easy to overcome.

**3) Programming power**: the power is consumed by setting the phase shifter of programmable mesh, so the programming power is proportional to the number of needed phase shifters. Usually, the phase value range in [0, 2π), therefore the consumed power for shifter-setting is more or less on the order of Pπ, which corresponds to the power to produce π-shift, thus the overall programming power can be estimated as ~2N^2^Pπ for SVD-based GridNet mesh, ~N(N-1)Pπ for pseudo-real GridUnitary mesh and ~Nlog_2_NPπ for pseudo-real FFTUnitary mesh, respectively. Hence even using more efficient shifter with Pπ~1 mW or less[[13-15](#_ENREF_13)], the programming power of conventional SVD-based GridNet mesh (pseudo-real GridUnitary mesh) at the radix of N=1024 would be still as high as ~2kW (1kW), while the pseudo-real FFTUnitary mesh becomes very energy efficient, requiring ~11W power.

**4) Encoding error**: in realistic MZI mesh, there are noises on the phase shifters resulting in the deviation of the encoded matrix from the ideal one. Such encoding error is optical depth dependent[[16](#_ENREF_16)], thus scales linearly with matrix dimension N as O(2N+1) in SVD-based GridNet mesh and O(N) in pseudo-real GridUnitary mesh, while only logarithmically as O(log_2_N) in pseudo-real FFTUnitary mesh. This might be why the performance of the PNN implemented on our pseudo-real unitary mesh could be very close to the model trained on digital computer, while the counterpart realized on SVD-mesh often exhibit apparent accuracy-loss probably due to the larger errors brought by larger optical depth[[17](#_ENREF_17)].

**References**

1. Mathieu, M. and Y. Lecun, *Fast Approximation of Rotations and Hessians matrices.* Computer Science, 2014.

2. Raz, R., *On the Complexity of Matrix Product.* siam journal on computing, 2003. **32**(5): p. 1356-1369.

3. Dasgupta, S. and A. Gupta, *An elementary proof of a theorem of Johnson and Lindenstrauss.* random structures and algorithms, 2003. **22**(1): p. 60-65.

4. Likhosherstov, V., K. Choromanski, and A. Weller, *On the Expressive Power of Self-Attention Matrices*, 2021.

5. Zhang, H., et al., *An optical neural chip for implementing complex-valued neural network.* Nature Communications, 2021. **12**(1): p. 457.

6. Fang, M.Y.S., et al., *Design of optical neural networks with component imprecisions.* Optics Express, 2019. **27**(10): p. 14009-14029.

7. Xue, W., et al., *Optoelectronic memristor for neuromorphic computing.* Chinese Physics B, 2020. **29**(4): p. 048401.

8. Gattass, R.R. and E. Mazur, *Femtosecond laser micromachining in transparent materials.* Nature Photonics, 2008. **2**(4): p. 219-225.

9. Shen, Y., et al., *Deep learning with coherent nanophotonic circuits.* Nature Photonics, 2017. **11**(7): p. 441-446.

10. Harris, N.C., et al., *Efficient, compact and low loss thermo-optic phase shifter in silicon.* Optics Express, 2014. **22**(9): p. 10487-10493.

11. Feldmann, J., et al., *Parallel convolutional processing using an integrated photonic tensor core.* Nature.

12. Carolan, J., et al., *Universal linear optics.* Science, 2015. **349**(6249): p. 711-716.

13. Totović, A.R., et al., *Femtojoule per MAC Neuromorphic Photonics: An Energy and Technology Roadmap.* IEEE Journal of Selected Topics in Quantum Electronics, 2020. **26**(5): p. 1-15.

14. Chung, S., M. Nakai, and H. Hashemi, *Low-power thermo-optic silicon modulator for large-scale photonic integrated systems.* Optics Express, 2019. **27**(9): p. 13430-13459.

15. Dong, P., et al., *Submilliwatt, ultrafast and broadband electro-optic silicon switches.* Optics Express, 2010. **18**(24): p. 25225-25231.

16. Zhang, X.M. and M.H. Yung, *Low-Depth Optical Neural Networks.* 2019.

17. Clements, W.R., et al., *Optimal design for universal multiport interferometers.* Optica, 2016. **3**(12): p. 1460-1465.
